# Constant and seasonal drivers of bird communities in a wind farm: implications for conservation

**DOI:** 10.7717/peerj.2105

**Published:** 2016-07-19

**Authors:** Zuzanna M. Rosin, Piotr Skórka, Paweł Szymański, Marcin Tobolka, Andrzej Luczak, Piotr Tryjanowski

**Affiliations:** 1 Department of Cell Biology, Adam Mickiewicz University in Poznań, Poznań, Poland; 2 Institute of Nature Conservation, Polish Academy of Sciences, Kraków, Poland; 3 Department of Behavioural Ecology, Adam Mickiewicz University in Poznań, Poznań, Poland; 4 Institute of Zoology, Poznań University of Life Sciences, Poznań, Poland; 5 ENINA, Poznań, Poland

**Keywords:** Agricultural landscape, Biodiversity, Field, Season, Settlement, Turbine

## Abstract

**Background.** One of the most difficult challenges for conservation biology is to reconcile growing human demands for resources with the rising need for protecting nature. Wind farms producing renewable energy have been recognised to be a threat for birds, but clear directives for environmental planning are still missing.

**Methods.** Point counts were performed to study the relationship between eight environmental variables and bird populations in different parts of a year on the largest Polish wind farm between March 2011 and February 2013. Variables potentially related to species richness (Chao 1 estimator) and the abundance of the entire bird community as well as five selected farmland species were analysed with the use of generalized linear mixed models.

**Results.** Some associations between the studied variables and bird populations were season/year specific, while others had a constant direction (positive or negative) across seasons and/or years. The latter were distance to the nearest turbine, field size, number of wind turbines, proximity of settlements and water bodies. Spatial autocorrelation and counting time were significantly correlated with bird population estimates but the directions of these relationships varied among seasons and years. Associations between abundance of individual species and environmental variables were species-specific.

**Conclusions.** The results demonstrated a constant negative relationship between wind turbine proximity and bird numbers. Other environmental variables, such as field size, proximity of settlements and water bodies that also had constant associations with bird populations across seasons may be taken into account when minimizing adverse effects of wind farm development on birds or choosing optimal locations of new turbines.

## Introduction

Human—wildlife conflicts are one of the main problems worldwide resulting from growing energy demand, agricultural intensification and expansion of urban areas on the one side, and rising needs for halting biodiversity loss, protecting natural ecosystems and sustaining their services on the other (Sovacool, 2013; Bakken et al., 2014; Little et al., 2014). These conflicts are the most serious in cases where investments designed to improve quality of the environment constitute a danger for ecosystem services including biodiversity, pest control and nutrient circulation (Leddy, Higgins & Naugle, 1999; Pearce-Higgins et al., 2012; Bakken et al., 2014). Thus, land management of habitats and landscapes should focus on finding solutions that will alleviate human—wildlife conflicts (Cole & Dahl, 2013; Miller et al., 2014; Mouysset, Doyen & Jiquet, 2014).

A good example of investments that have both costs and benefits to the environment is wind farms—carbon-free power stations that have mostly negative direct and indirect effects on animals, particularly birds and bats (e.g., Drewitt & Langston, 2006; Kuvlesky et al., 2007; Santone et al., 2013). According to some authors (Drewitt & Langston, 2006; Kuvlesky et al., 2007) these effects include collisions with turbines, displacement due to disturbance, alterations of habitat and microclimate, and also consequences for vocal communication (Zwart et al., 2016). Several groups of birds, for example farmland birds, have recently been considered one of the most endangered vertebrates in Europe (e.g., Krebs et al., 1999; Tryjanowski, 2000; Tryjanowski et al., 2011). Hence, to effectively minimize the adverse effects of wind farms on birds, it is essential to recognize the factors that influence species richness and abundance of birds within farms (Marques et al., 2014). The majority of studies on wind farm-bird relationships focused on describing the negative effects of turbines on bird populations in one period of the annual cycle of birds, usually migration or breeding (e.g., Leddy, Higgins & Naugle, 1999). However, the effects of different environmental variables may vary during a year and among years (e.g., Carrete et al., 2012), which may generate difficulties in the management of habitats within wind farms in a constant conservation-friendly way for birds. Therefore, the crucial problem is to identify these factors that have consistent effect on birds within a wind farm regardless of the season and year. To date this difficulty has not been overcome in wind farms despite their growing importance for world energy markets (Trancik, 2014) and rising evidence of their negative effects on birds (Kuvlesky et al., 2007).

The aim of this study was to determine the factors that have significant predictive relationships with bird species richness and abundance in the same direction among seasons and years, to provide practical guidelines for the management and development of a wind farm. We expected constant negative relations of wind turbines vicinity with bird species richness and abundance (according to Drewitt & Langston, 2006; Kuvlesky et al., 2007) that however may be modified by local environmental conditions (e.g., presence of water bodies, settlements, high fields diversity) as well as undergo seasonal variations. Moreover, we also studied associations between abundance of selected common farmland species and environmental variables to test whether response of individual species is representative for the entire bird community.

## Methods

### Study area

The study was conducted in the largest Polish wind farm located in Wielkopolska Province near Margonin (52°58′N, 17°05′E) between 2011 and 2013. The wind farm was composed of 60 turbines (currently 113 turbines) with total power of 120 MW and covered an area exceeding 100 km^2^. The windmills work since 2009 and are characterized by tower height of 100 m and blade span of 90 m. The wind farm is located in intensive agricultural landscape composed mainly of arable fields (cereals, potatoes, maize) diversified by forests and water basins.

### Data collection

At the study plot, 36 points were randomly selected from a regular grid of 0.5 km squares. Bird surveys for this study were obtained by counting birds once a ten days between March 2011 and February 2013. Standard bird point counts (Bibby et al., 2000; Sutherland, Newton & Green, 2004), lasting 5 min, with a 250-m sampling radius were conducted at each point by two experienced observers (PSz, MT) beginning at dawn and conducted until 4.5 h after sunrise. Each visit was conducted during favourable weather conditions without rain and little or no wind (below 3 in the Beafourt scale). Observers moved between points by cars. The counts were performed using binoculars (10 × 42); numbers of all seen and heard individuals of each species were recorded. The study was conducted in accordance with Polish law.

The following environmental explanatory variables potentially affecting the species richness and abundance of birds were calculated for each counting point in Quantum GIS 1.7 for each point (Table 1):

**Table 1:** Basic summary statistics of explanatory and dependent variables in randomly selected points within the studied wind farm (*n* = 36). Chao 1 estimates are given in brackets.

Variable	Abbreviation	Mean ± SE	Min	Max
***Explanatory variables***				
Mean area of agriculture fields within a 1-km radius (ha)	FieldS	13.9 ± 1.9	2.4	44.4
Index of field area diversity within a 1-km radius	CV	0.733 ± 0.050	0.184	1.533
Road length within a 1-km radius (m)	Road	7,569 ± 348	2,814	12,611
Forest cover (%) within a 1-km radius (ha)	Forest	21.6 ± 4.3	0.2	93.6
Distance to the nearest human settlements (m)	Settle	353 ± 40	45	1,129
Distance to the nearest water basin (m)	Water	640 ± 74	99	1,909
Distance to the nearest wind turbine (m)	DTurb	398 ± 42	20	998
Number of wind turbines in a 1-km radius	NTurb	3.2 ± 0.3	1	7
***Dependent variables***				
***Species richness : periods & years(Chao 1 estimator in brackets)***				
Spring migration (March–April) 2011		6.4 ± 0.2 (9.5 ± 0.6)	0 (0)	15 (47)
Spring migration (March–April) 2012	–	7.1 ± 0.2 (9.2 ± 0.4)	1 (1)	16 (35)
Breeding season (May–July) 2011		7.4 ± 0.2 (11.9 ± 0.5)	1 (1)	19 (83)
Breeding season (May–July) 2012		8.6 ± 0.2 (13.5 ± 0.6)	1 (1)	19 (107)
Autumn migration (August–November) 2011		4.9 ± 0.1 (6.2 ± 0.2)	0 (0)	19 (36)
Autumn migration (August–November) 2012		5.8 ± 0.2 (7.6 ± 0.3)	0 (0)	19 (41)
Winter (December–February) 2011		2.0 ± 0.1 (2.4 ± 0.2)	0 (0)	7 (16)
Winter (December–February) 2012		2.8 ± 0.1 (3.2 ± 0.2)	0 (0)	10 (16)
***Abundance: periods & years***				
Spring migration (March–April) 2011		35.3 ± 7.5	0	1,233
Spring migration (March–April) 2012		26.1 ± 3.7	5	619
Breeding season (May–July) 2011		25.4 ± 2.8	2	766
Breeding season (May–July) 2012	–	25.1 ± 1.2	2	178
Autumn migration (August–November) 2011		42.3 ± 3.1	0	633
Autumn migration (August–November) 2012	–	40.5 ± 3.7	0	965
Winter (December–February) 2011		9.3 ± 1.1	0	134
Winter (December–February) 2012	–	17.0 ± 2.4	0	283

1. Mean area (ha) of agricultural fields within a 1-km radius. Generally, larger crop fields are known to reduce bird species richness (Morales et al., 2012), especially in intensively managed landscape as it was the case in our study area.
2. Variation in field area measured as the coefficient of variation in a 1-km radius. Different farmland species have various habitat preferences in terms of field size (e.g., Rosin et al., 2012; Altewischer et al., 2015) thus higher variation in field size may increase spatial heterogeneity and thus species richness (Pickett & Siriwardena, 2011).
3. Road length (m) within a 1-km radius. Asphalt and country roads were included. Major effect of road on birds is mortality via collisions with cars thus one may expect negative relationship between road density and species richness (Orłowski, 2008; Mammides, Kadis & Coulson, 2015).
4. Forest cover (%) within a 1-km radius (ha). Woodland patches may serve as a breeding habitat for some species foraging in farmland and thus may increase total species richness (Tworek, 2004; Bouvier et al., 2014).
5. Distance (m) to the nearest human settlements. Human settlements, usually belonging to farmers are breeding habitat for some farmland species (e.g., sparrows, swallows) and thus may increase species richness (Šálek et al., 2015). However, humans may also negatively affect wild species by disturbance and activity of domestic animals (Rosin et al., 2012; Doherty, Bengsen & Davis, 2014).
6. Distance (m) to the nearest water body (a pond or lake larger than 1 ha). Former study indicated that water habitats increase farmland biodiversity (Surmacki, 1998).
7. Distance (m) to the nearest wind turbine. This variable describes impact of the nearest wind turbine on birds.
8. Number of wind turbines within a 1-km radius. This variable was calculated because it described joint impact of a few turbines on birds.

### Data handling and analysis

Our dependent variables were species richness and abundance of birds in every survey. Since the inherent feature of field survey is imperfect detection of species, we used corrected number of species by calculation Chao-1 estimator for each survey (Gotelli & Chao, 2013). Non-parametric estimators (e.g., Chao1) perform best in empirical comparisons and benchmark surveys, and have a more rigorous framework of sampling theory (demonstrating that both are robust estimators of minimum richness; Shen, Chao & Lin, 2003) than other methods, e.g., parametric estimators (Gotelli & Chao, 2013).

Generalized linear mixed models with Poisson error and log-link function were built to analyze the factors affecting Chao 1 species richness estimator and abundance in each survey during following seasons of the year that relate to biological cycles of bird life; (1) spring migration (March–April), (2) breeding period (May–July), (3) autumn dispersal and migration (August–November), and (4) winter (December–February). The “lme4” package (Bates et al., 2015) implemented in R (R Core Team, 2014) was used for this purpose. Models were built separately for every season in 2011 (March 2011–February 2012) and 2012 (March 2012–February 2013). To take spatial autocorrelation in dependent variables for each survey into account, we applied the approach proposed by Diniz-Filho, Rangel & Bini (2008). Spatial autocovariate was calculated in the SAM 4.0 statistical software (Rangel, Diniz-Filho & Bini, 2010). The spatial autocorrelation was modelled as an autoregressive term in generalized linear mixed models. Thus, our model was a lagged-response model (Dormann et al., 2007). The spatial term was present in every model. We also included time of the survey started at each point as a covariate. The time was expressed as minutes from the sunrise of local time and it was calculated with the help of on-line calculator (http://darekk.com/sunrise-sunset-calculator). Both linear and quadratic terms were included in the model because the activity of birds may change in a non-linear way since sunrise. Thus, independent variables used in modelling were all environmental variables (1)–(8) described above, spatial autocovariate and time since sunrise (both linear and quadratic terms). We also included three random factors: observer, counting point ID and survey identities.

The analogous models were built for the abundance of five selected common species typical for farmland (skylark *Alauda arvensis*, yellowhammer *Emberiza citrinella*, yellow wagtail *Motacilla flava*, lapwing *Vanellus vanellus* and common whitethroat *Sylvia communis*) to investigate if their response to environmental factors differed from the response of the entire bird assemblage.

For each dependent variable and for each period we built all possible model combinations. For abundance of five individual species, all possible model combinations were also built except periods in which there were less than two observations of a given species. Akaike’s Information Criterion (AICc) was calculated for each possible model with the help of “MuMIn” package (Bartoń, 2014). We ranked models according to their ΔAICc values and used the model with the lowest AICc together with associated weight value (the probability that a given model is the best) as that best describing the data. We considered models with ΔAICc lower than 2 as equally good (Burnham & Anderson, 2002). We used model averaging for estimates of function slopes of parameters of interest (Burnham & Anderson, 2002). For model averaging we used 95% confidence set (we used all models with a sum of weights equalling 0.95).

When necessary, we used square root transformation of environmental variables to reduce the effects of outlier observations (Quinn & Keough, 2002). Moreover, in all regression models, variables were standardized to allow a direct comparison of beta (slope) estimates (larger values of betas indicate stronger relationships between explanatory and dependent variables). All variables included in the analyses were weakly correlated between each other (|*r*| < 0.427, Table S1). Regression models are robust to multicollinearity if the correlation between variables is lower than *r* = 0.6 (Mertler & Vannatta, 2002).

All estimates of statistical parameters (means, slopes) are quoted with standard errors (SE) and/or 95% confidence intervals (CI). We considered the slopes of the generalized linear mixed model function to be significant if their 95% CI did not overlap with zero.

We considered an association between explanatory and dependent variable as constant if there was at least one significant result or if all significant relationships recorded for studied seasons had the same direction (the estimates of function slopes were constantly positive or negative during different seasons).

## Results

### Summary statistics

Altogether 39,443 individual birds belonging to 128 species were observed between March 2011 and February 2012, and 38,361 individuals of 116 species between March 2012 and February 2013 (Table S2). The highest numbers of birds occurred during autumn migration and breeding season (Table 1). The lowest numbers were noted in winter (Table 1). Mean values for explanatory variables and correlations among them are given in Table 1 and Table S1, respectively.

### Bird species richness

**Figure 1:**
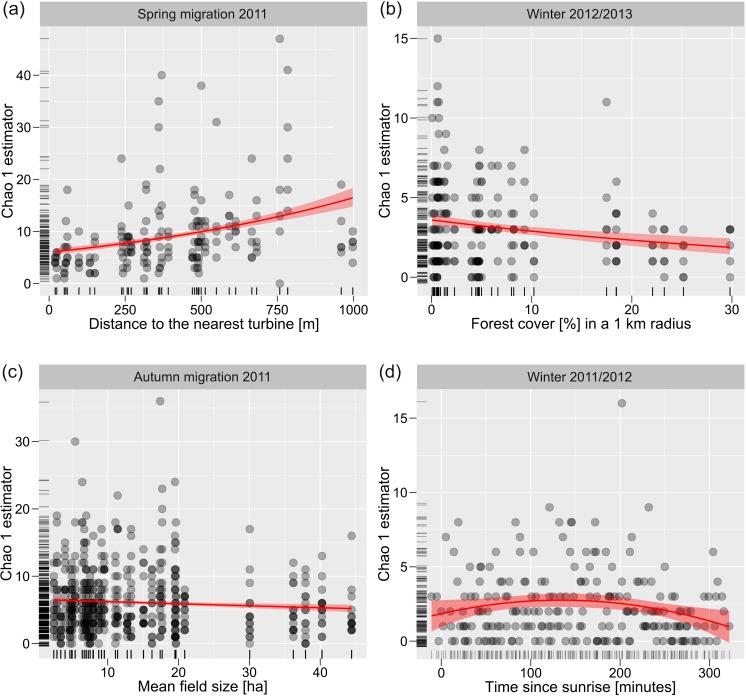
Relationships between bird species richness and environmental variables. Exemplary relationships between bird species richness and distance to the nearest turbine (A), forest cover in a 1-km radius (B), mean field size (C), and quadratic effect of time since sunrise (D). Poisson regression fitted with standard errors.

The strength and direction of relationships between Chao 1 species richness estimator and the studied environmental variables varied depending on period and year (Table 2, Tables S3 and S4). Constant direction across periods of the year and between years had correlations between species richness estimator and distance to the nearest turbine (positive, Fig. 1, Table 2 and Table S4), mean field size (negative, Fig. 1, Table 2 and Table S4) and spatial autocovariate (positive, that indicated that species richness was spatially predictable; Table 2 and Table S4). Also, constant association was found for time on the day of the survey with decreasing number of birds since sunrise (Table 2 and Table S4). Forest cover positively correlated with Chao 1 species richness estimator in breeding period 2012, but negatively in winter 2012/2013 (Fig. 1. Table 2, Table S4). There was a non-linear relationship between the estimates of species richness and time since sunrise (Table 2 and Table S4). Generally, the number of species increased for about two hours since sunrise, and later it decreased (Fig. 1) but not in breeding period 2012 (Table 2 and Table S4).

**Table 2:** Summarized effects (function slopes ± (SE)) of variables correlated to Chao 1 estimator of species richness and abundance in the studied wind farm in different seasons and years. Orange cells indicate statistically significant positive relationships, blue cells indicate statistically significant negative relationships and grey cells indicate non-significant relationships.

	**Explanatory variables**										
	Auto	CV	DTurb	FieldS	Forest	NTurb	Road	Settle	Sunrise	Sunrise^∧^2	Water
**Chao 1 estimator**											
Spring migration 2011			0.285 (0.085)						−0.067 (0.034)	−0.173 (0.039)	
Spring migration 2012	0.148 (0.037)		0.199 (0.071)						−0.085 (0.027)		
Breeding period 2011	0.027 (0.012)								−0.169 (0.023)		
Breeding period 2012	0.100 (0.029)		0.185 (0.092)		0.188 (0.077)					0.121 (0.032)	
Autumn migration 2011	0.049 (0.013)		0.187 (0.051)						−0.140 (0.019)		
Autumn migration 2012	0.629 (0.084)		0.160 (0.048)	−0.168 (0.047)						−0.054 (0.027)	
Winter 2011/2012	0.136 (0.029)		0.427 (0.096)							−0.248 (0.085)	
Winter 2012/2013	0.359 (0.063)				−0.167 (0.076)						
***Consistency***	+		+	−					−		
**Abundance**											
Spring migration 2011	0.111 (0.015)	0.250 (0.114)	0.262 (0.133)						−0.126 (0.024)	−0.423 (0.023)	
Spring migration 2012	0.414 (0.068)								−0.326 (0.017)	0.075 (0.021)	
Breeding period 2011	−0.227 (0.015)	−0.159 (0.079)						−0.192 (0.094)	0.038 (0.017)	0.047 (0.011)	
Breeding period 2012	0.241 (0.042)							-0.169 (0.072)		0.050 (0.013)	
Autumn migration 2011	−0.054 (0.008)	−0.161 (0.071)							−0.154 (0.007)	0.270 (0.011)	−0.174 (0.075)
Autumn migration 2012	2.310 (0.036)							−0.254 (0.095)	−0.068 (0.007)	0.036 (0.013)	
Winter 2011	−0.807 (0.040)	−0.345 (0.153)	0.344 (0.172)	−0.364 (0.165)					−0.097 (0.027)		
Winter 2012	2.248 (0.101)	0.314 (0.136)				0.345 (0.151)			−0.907 (0.017)	−0.263 (0.028)	
***Consistency***			+	−		+		−			−

### Abundance of birds

**Figure 2:**
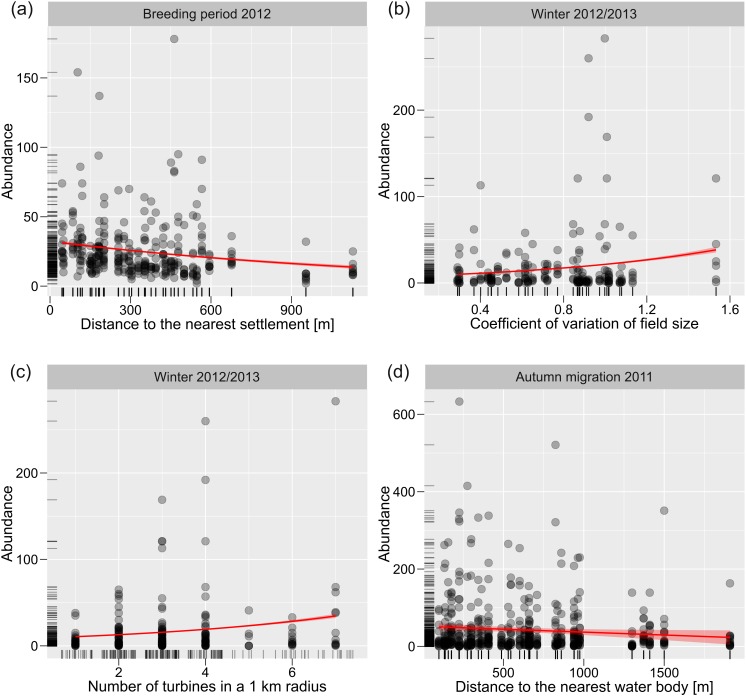
Relationships between bird abundance and environmental variables. Exemplary relationships between bird abundance and distance to the nearest settlements (A), variation in field size (B), number of wind turbines in a 1-km radius (C), and distance to the nearest water body (D). Poisson regression fitted with standard errors.

Similarly to species richness, the strength and direction of relationships found between species abundance and environmental variables depended on season and year (Table 2, Tables S5 and S6). Among the variables correlated with bird abundance the constant association across periods of the year and among years was found for distance to the nearest turbine (positive; Table 2 and Table S6), distance to the nearest settlement and water body (negative, Fig. 2, Table 2 and Table S6) and number of turbines in a 1-km radius (positive Fig. 2, Table 2 and Table S6). Variation in field size positively correlated with abundance of birds during spring migration 2011 and in winter 2012/2013 (Fig. 2, Table 2 and Table S6), but negatively during breeding period 2011, autumn migration 2011 and winter 2011/2012 (Table 2 and Table S6). There were statistically significant, both linear and non-linear, relationships between bird abundance and time since sunrise, but directions of these relationships varied (Table 2 and Table S6).

### Individual species responses

Relationships between abundance of selected species and environmental variables varied in strength and direction depending on species. Skylark abundance was constantly related to forest cover (negative), spatial autocovariate (positive), distance to the nearest settlement (positive) and water body (negative, Table 3 and Table S8). There were constant associations between abundance of yellowhammer and field area diversity (positive), distance to the nearest turbine and forest cover (positive, Table 3 and Table S10). Abundance of yellow wagtail was constantly related to distance to the nearest turbine (negatively), forest cover (negatively), number of turbine (positively), distance to the nearest settlement (positively) and water basin (negatively, Table 3 and Table S12). Lapwing abundance was constantly related to forest cover (negatively) and number of turbines (negatively, Table 3 and Table S14). There were constant associations between abundance of common whitethroat and distance to the nearest turbine (positive), and road length within a 1-km radius (positive, Table 3 and Table S16). There were statistically significant, both linear and non-linear, relationships between species abundance and time since sunrise, and they were constant across seasons in case of yellowhammer and yellow wagtail (negative, Table 3, Tables S10 and S12).

**Table 3:** Summarized effects (function slopes ± (SE)) of variables correlated to abundance of selected farmland bird species in the studied wind farm in different seasons and years. Orange cells indicate statistically significant positive relationships, blue cells indicate statistically significant negative relationships and grey cells indicate non-significant relationships.

Season	**Explanatory variables**										
	Auto	CV	DTurb	FieldS	Forest	NTurb	Road	Settle	Sunrise	Sunrise^∧^2	Water
**Skylark *Alauda arvensis***											
Spring migration 2011	0.433 (0.176)					0.117 (0.041)		0.155 (0.041)			−0.143 (0.042)
Spring migration 2012	0.421 (0.083)				−0.083 (0.042)		−0.094 (0.041)	0.085 (0.039)			−0.096 (0.041)
Breeding period 2011	0.615 (0.080)				−0.120 (0.056)			0.181 (0.050)			
Breeding period 2012	0.580 (0.109)				−0.086 (0.040)						−0.099 (0.038)
Autumn migration 2011	2.294 (0.310)				−0.645 (0.165)				0.555 (0.74)	−0.450 (0.107)	
Autumn migration 2012	1.698 (0.367)								0.596 (0.100)	−0.858 (0.123)	
**Consistency**	+				−	+	−	+	+	−	−
**Yellowhammer *Emberiza citrinella***											
Spring migration 2011		0.533 (0.218)			0.823 (0.268)						
Spring migration 2012	−0.289 (0.123)		0.344 (0.149)								
Breeding period 2011	−0.344 (0.108)		0.284 (0.139)		0.417 (0.142)						
Breeding period 2012					0.352 (0.128)				−0.156 (0.061)		
Autumn migration 2011	0.306 (0.045)	0.276 (0.128)			0.433 (0.129)				−0.161 (0.040)		
Autumn migration 2012	0.164 (0.060)								−0.167 (0.051)	−0.408 (0.090)	
Winter 2011	5.362 (0.429)										
Winter 2012	0.347 (0.146)									−0.418 (0.100)	
**Consistency**		+	+		+				−	−	
**Yellow wagtail *Motacilla flava***											
Spring migration 2011	−0.448 (0.171)			−0.163 (0.002)	−0.313 (0.002)	0.368 (0.107)		0.258 (0.008)	−0.353 (0.133)	−0.361 (0.183)	−0.387 (0.104)
Spring migration 2012											
Breeding period 2011	−0.483 (0.238)		−0.225 (0.094)	0.266 (0.090)	−0.445 (0.105)					−0.119 (0.059)	
Breeding period 2012		−0.242 (0.119)			−0.279 (0.127)			0.308 (0.121)			
Autumn migration 2011									−0.593 (0.083)		-0.600 (0.125)
Autumn migration 2012	0.718 (0.316)		−0.284 (0.121)			0.248 (0.126)	−0.371 (0.136)		−0.277 (0.104)	−0.374 (0.120)	−0.297 (0.129)
**Consistency**		−	−		−	+	−	+	−	−	−
**Lapwing *Vanellus vanellus***											
Spring migration 2011		−3.717 (1.255)		1.971 (0.718)		−2.527 (1.036)					−1.508 (0.555)
Spring migration 2012									−0.949 (0.476)		
Breeding period 2011					−1.615 (0.740)	−3.114 (0.974)					
Autumn migration 2011					−30.247 (7.892)		12.893 (4.371)		0.778 (0.219)	36.350 (3.830)	
**Consistency**		−		+	−–	−	+			+	−
**Common whitethroat *Sylvia communis***											
Spring migration 2011											
Spring migration 2012											
Breeding period 2011			0.383 (0.167)								
Breeding period 2012			0.317 (0.142)			−0.091 (0.018)	0.364 (0.119)	−0.305 (0.104)		0.257 (0.091)	
Autumn migration 2011											
Autumn migration 2012							2.088 (0.766)				
**Consistency**			+			−	+	−		+

## Discussion

In this study we show that the strength and direction of relationships between environmental variables and bird species richness and abundance in a wind farm vary greatly among seasons and years. Novelty of our paper lays in determining the variables that had significant, constant and predictive relationships with bird communities.

Avian biology and habitat requirements in temperate regions substantially vary depending on part of a year (season). Thus, the effect of environmental factors on birds changes within and between years (Møller, 1984; Carrete et al., 2012). This obvious fact has serious consequences for working out efficient conservation strategies. For example, in our study, forest cover within a 1-km radius from a wind turbine was positively related with species richness during breeding period 2012 but negatively correlated with species richness during winter 2012/2013. Therefore, the environmental variables that are related to bird populations in one direction (positive or negative) across seasons and years are the most valuable for conservation of biodiversity in wind farms (and possibly other areas). Implementing conservation actions usually assumes constant and predictable effects of some alterations in the environment on species. For example, planting trees or creating land-use mosaic assume their constant effect on species richness and abundance. Of course, the factors appearing seasonally or stochastically may have also a considerable impact on birds in wind farms. However, if a given environmental variable affects species richness and abundance in different directions among seasons and/or years, then conservation actions altering that variable would be a waste of money and resources. These remarks raise concerns about the suitability and unknown consequences of studies conducted in a specific period (e.g., breeding season) or only a single year.

Among the variables that showed consistent patterns over time was distance to the nearest turbine that positively correlated with species richness and abundance. This is one of the first pieces of evidence that proximity of wind turbines is negatively related to bird communities within wind farms (Leddy, Higgins & Naugle, 1999). The direct effect of turbines, namely moving blades, may chase or kill birds. Wind turbines may also possibly change birds’ movement pattern across the landscape (Kuvlesky et al., 2007). We also observed that during cold days, mostly in winter, blades are covered by ice. In the middle of a day, when temperature rises, this ice drops and is scattered by blades in the proximity of turbines (P Skórka, 2013, unpublished data). As ice particles are numerous and often fairly large, they may directly negatively affect bird behaviour. Moreover, Zwart et al. (2016) showed experimentally that in the presence of wind farm noise, male robins *Erithacus rubecula* substantially reduced usage of low-frequency elements during simulated territorial intrusions. This is particularly important in a context of social interactions between males from adjacent territories, since low-frequency songs are used in many song birds in territory defense as a signal of aggressive motivation and threatening display (Benedict, Rose & Warning, 2012). This, in turn, may lead to difficulties in territory holding (McMullen, Schmidt & Kunc, 2014; Zwart et al., 2016). Unexpectedly, in one season (winter 2012/2013), we observed positive association between abundance of birds and number of turbines within a 1-km radius. Anthropogenic structures, for example electricity pylons, are associated with specific microhabitats that may contribute to conservation of birds in intensive farmland (Tryjanowski et al., 2014). Under turbines, there are sites with ruderal vegetation, puddles and singular stubs, all creating potentially suitable foraging or breeding microhabitats for birds. Moreover, wind turbines attract invertebrates that may constitute food for birds (Dudek et al., 2016). These factors may be responsible for occasional positive relationship between birds and wind turbines, and counterbalance the negative impact of the latter. Our study did not consider mortality of birds within a farm as we found only six carcasses, which did not allow for reasonable analysis. Thus, the impact of the studied variables on turbine-related mortality of birds should be investigated in future studies.

Abundance of birds decreased with increasing distance to the nearest settlements and water basins. Many farmland birds are actually synanthropic species (e.g., *Passer montanus, P. domesticus, Carduelis cannabina, C. chloris*) that benefit from human settlements and various buildings belonging to farms where they find food and breeding sites (e.g., Tryjanowski et al., 2009; Skórka et al., 2013; Rosin et al., in press). Also, several species breeding in human settlements, such as barn swallow *Hirundo rustica*, common starling *Sturnus vulgaris* and white wagtail *Motacilla alba*, usually perform foraging trips to the agricultural fields located in the proximity of buildings. The effect of distance to water was significant for abundance in autumn migration in 2012. Water bodies in intensive farmland are refuges for insects (Ruggiero et al., 2008; Raebel et al., 2012) and have specific microhabitats that attract farmland birds (Surmacki, 1998). Moreover, this habitat is frequently used during spring and autumn migration, especially by geese and waders (e.g., Rosin et al., 2012).

Field size was negatively correlated to species richness and abundance during two seasons. This result is in agreement with other studies showing that agricultural landscapes with smaller fields are inhabited by more species (e.g., Söderström & Pärt, 2000; Herzon & O’Hara, 2007). Fields in Poland are separated from each other by narrow grassy strips, thus smaller fields increase the density of these potentially good microhabitats for birds (Concepción et al., 2012). Therefore, another initiative that could help to maintain species richness of birds in wind farms is to divide large fields into smaller ones. Alternatively, if the goal is to reduce the impact of wind turbines on birds, then joining smaller fields into larger ones may hinder birds from settling in the area of wind farms.

Among the variables that showed inconsistent patterns across seasons, coefficient of variation in field area was especially interesting. This variable was positively associated with bird abundance in winter 2011/2012 but negatively in the following winter, and there were no significant relationships with species richness in any examined season. This result contributes to the latest suggestions and findings that relationship between diversity of crop type and size and bird diversity is not always obvious and positive (e.g., Batáry et al., 2011; Hiron et al., 2015). Similar varying effect was found for forest cover.

Relationships between environmental variables and abundances of selected farmland bird species were species-dependent and varied across seasons and years but were in agreement with the biology of these species. Abundances of yellowhammer and common whitethroat were constantly negatively related to proximity of turbines, and this is consistent with the result for the entire bird community. However, abundance of yellow wagtail increased with decreasing distance to the nearest turbine, possibly because of presence of ruderal vegetation around the base of turbines. Other season-consistent associations were in agreement with habitat preferences of individual species. For example, skylark, yellow wagtail and lapwing were negatively associated with forest cover, whereas yellow wagtail preferred the vicinity of water basins (compare: Snow & Perrins, 1998).

## Conclusions

The effect of wind farms on birds may have a strong local component (Drewitt & Langston, 2006; Marques et al., 2014). Nevertheless it is desirable to determine a universal set of environmental drivers of biodiversity within farms because they become more and more common in agricultural landscapes of Europe. Our study provides new perspectives on conservation of birds in wind farms, comparing to earlier studies (e.g., Carrete et al., 2012; Miller et al., 2014). We recommend long-term (at least two-year) studies for pre-construction evaluation to determine the factors that influence bird communities in a predictable way regardless of season and year. Negative effects of a wind farm on birds may be reduced by appropriate location of wind turbines (large fields, large distance to settlements and water basins) or by creating/altering specific microhabitats within farms.

##  Supplemental Information

10.7717/peerj.2105/supp-1 Table S1 Correlations between explanatory environmental variable Correlations between explanatory environmental variables. *P* values (in brackets) are corrected to take spatial autocorrelation into account. Statistically significant relations are bolded. Explanations of variable abbreviations are given in Table 1.

10.7717/peerj.2105/supp-2 Table S2 List of all species observe List of all species observed in the wind farm during seasons of 2012 and 2013. Sum of individuals is given.

10.7717/peerj.2105/supp-3 Tables S3–S16 Best models and averaged values of function parameters Best models and averaged values of function parameters of variables correlated to Chao 1 bird species richness estimator, abundance of entire bird community, and abundance of five selected farmland species in different seasons and years.

10.7717/peerj.2105/supp-4 Data S1 Raw data

## Additional Information and Declarations

### Data Availability

The following information was supplied regarding data availability: The raw data has been supplied as Data S1.
