# Supplementary material for: Constant and seasonal drivers of bird communities in a wind farm: implications for conservation
Source: PeerJ. 2016 Jul 19;4:e2105. doi: 10.7717/peerj.2105 (PMC4957985; doi:10.7717/peerj.2105)
Supplement: Table S1 — Correlations between explanatory environmental variables. P values (in brackets) are corrected to take spatial autocorrelation into account. Statistically significant relations are bolded. Explanations of variable abbreviations are given in Table 1. [file peerj-04-2105-s001.docx]

**SUPPLEMENTARY MATERIAL**

Table S1. Correlations between explanatory environmental variables. P values (in brackets) are corrected to take spatial autocorrelation into account. Statistically significant relations are bolded. Explanations of variable abbreviations are given in Table 1.

|  | Fields | CV | Road | Forest | Settle | Water | DTurb | NTurb |
| --- | --- | --- | --- | --- | --- | --- | --- | --- |
| FieldS | - | 0.010 (0.958) | -0.093 (0.662) | 0.083 (0.608) | 0.324 (0.080) | 0.021 (0.909) | 0.189 (0.239) | 0.218 (0.403) |
| CV |  | - | -0.048 (0.802) | -0.054 (0.792) | -0.006 (0.977) | -0.203 (0.238) | -0.004 (0.979) | -0.292 (0.167) |
| Road |  |  | - | -0.224 (0.211) | -0.306 (0.091) | -0.184 (0.283) | -0.090 (0.608) | 0.181 (0.368) |
| Forest |  |  |  | - | -0.002 (0.993) | **0.427 (0.013)** | **0.416 (0.011)** | -0.317 (0.070) |
| Settle |  |  |  |  | - | 0.079 (0.659) | **-0.372 (0.027)** | 0.105 (0.539) |
| Water |  |  |  |  |  | - | 0.162 (0.307) | 0.042 (0.806) |
| DTurb |  |  |  |  |  |  | - | 0.105 (0.539) |
| NTurb |  |  |  |  |  |  |  | - |
