# Supplementary material for: Constant and seasonal drivers of bird communities in a wind farm: implications for conservation
Source: PeerJ. 2016 Jul 19;4:e2105. doi: 10.7717/peerj.2105 (PMC4957985; doi:10.7717/peerj.2105)
Supplement: Table S2 — List of all species observed in the wind farm during seasons of 2012 and 2013. Sum of individuals is given. [file peerj-04-2105-s002.docx]

**SUPPLEMENTARY MATERIAL**

Table S2. List of all species observed in the wind farm during seasons of 2012 and 2013. Sum of individuals is given.

|  | Year 2011 | | | | Year 2012 | | | |
| --- | --- | --- | --- | --- | --- | --- | --- | --- |
| Species | Spring  migr. 2011 | Breed. period 2011 | Autumn migr. 2011 | Winter 2011 - 2012 | Spring  migr. 2012 | Breed. period 2012 | Autumn migr. 2012 | Winter  2012 - 2013 |
| *Sturnus vulgaris* | 512 | 2763 | 8965 | 0 | 224 | 1629 | 4915 | 0 |
| *Fringilla coelebs* | 166 | 55 | 2732 | 9 | 478 | 84 | 1699 | 1097 |
| *Anser sp.* | 2754 | 23 | 79 | 0 | 140 | 0 | 1346 | 527 |
| *Alauda arvensis* | 804 | 1369 | 456 | 2 | 814 | 1137 | 308 | 2 |
| *Passer montanus* | 157 | 434 | 970 | 479 | 223 | 866 | 2010 | 1242 |
| *Hirundo rustica* | 9 | 715 | 1101 | 0 | 39 | 874 | 1440 | 0 |
| *Emberiza calandra* | 206 | 364 | 719 | 404 | 232 | 506 | 619 | 341 |
| *Emberiza citrinella* | 80 | 149 | 541 | 531 | 112 | 272 | 357 | 438 |
| *Carduelis cannabina* | 33 | 64 | 521 | 205 | 61 | 105 | 109 | 99 |
| *Parus major* | 30 | 75 | 497 | 166 | 75 | 136 | 743 | 199 |
| *Columba palumbus* | 134 | 156 | 475 | 0 | 95 | 97 | 167 | 40 |
| *Larus ridibundus* | 112 | 97 | 393 | 0 | 362 | 157 | 27 | 0 |
| *Motacilla flava* | 51 | 333 | 202 | 0 | 24 | 334 | 143 | 0 |
| *Passer domesticus* | 40 | 91 | 357 | 79 | 76 | 150 | 176 | 24 |
| *Carduelis spinus* | 317 | 2 | 170 | 61 | 48 | 0 | 219 | 278 |
| *Vanellus vanellus* | 21 | 19 | 447 | 5 | 13 | 22 | 60 | 100 |
| *Corvus cornix* | 41 | 60 | 257 | 60 | 14 | 70 | 123 | 136 |
| *Columba oenas* | 19 | 7 | 94 | 278 | 15 | 38 | 61 | 276 |
| *Pica pica* | 77 | 64 | 117 | 132 | 62 | 70 | 147 | 92 |
| *Corvus corax* | 27 | 33 | 202 | 125 | 47 | 42 | 112 | 64 |
| *Grus grus* | 64 | 89 | 202 | 0 | 47 | 76 | 195 | 0 |
| *Carduelis carduelis* | 17 | 27 | 241 | 64 | 53 | 100 | 114 | 28 |
| *Anser fabalis* | 149 | 0 | 158 | 16 | 158 | 0 | 735 | 849 |
| *Motacilla alba* | 76 | 79 | 125 | 0 | 96 | 99 | 139 | 0 |
| *Carduelis chloris* | 20 | 16 | 102 | 112 | 31 | 45 | 268 | 430 |
| *Delichon urbica* | 1 | 161 | 67 | 0 | 10 | 177 | 116 | 0 |
| *Corvus monedula* | 2 | 2 | 193 | 31 | 8 | 3 | 202 | 51 |
| *Turdus pilaris* | 11 | 2 | 106 | 97 | 615 | 3 | 97 | 251 |
| *Buteo buteo* | 8 | 21 | 101 | 75 | 21 | 30 | 91 | 105 |
| *Cyanistes caeruleus* | 10 | 26 | 143 | 15 | 25 | 39 | 285 | 31 |
| *Sylvia communis* | 8 | 143 | 21 | 0 | 4 | 157 | 8 | 0 |
| *Corvus frugilegus* | 0 | 0 | 150 | 0 | 4 | 0 | 6 | 2 |
| *Galerida cristata* | 13 | 35 | 65 | 30 | 18 | 27 | 54 | 19 |
| *Ardea cinerea* | 27 | 82 | 14 | 0 | 5 | 27 | 3 | 0 |
| *Coccothraustes coccothraustes* | 4 | 41 | 26 | 40 | 7 | 23 | 47 | 7 |
| *Phoenicurus ochruros* | 24 | 35 | 48 | 0 | 24 | 39 | 22 | 0 |
| *Garrulus glandarius* | 15 | 4 | 70 | 17 | 6 | 14 | 85 | 13 |
| *Anas platyrhynchos* | 49 | 24 | 11 | 0 | 45 | 45 | 19 | 5 |
| *Circus aeruginosus* | 22 | 18 | 33 | 0 | 15 | 20 | 22 | 0 |
| *Streptopelia decaocto* | 4 | 24 | 26 | 13 | 11 | 27 | 64 | 32 |
| *Anser anser* | 4 | 19 | 39 | 2 | 12 | 7 | 45 | 8 |
| *Anthus pratensis* | 9 | 1 | 54 | 0 | 12 | 1 | 82 | 0 |
| *Sylvia atricapilla* | 9 | 50 | 5 | 0 | 9 | 67 | 14 | 0 |
| *Emberiza schoeniclus* | 19 | 20 | 21 | 3 | 18 | 22 | 22 | 1 |
| *Ciconia ciconia* | 15 | 31 | 11 | 0 | 15 | 17 | 14 | 0 |
| *Lanius collurio* | 0 | 36 | 18 | 0 | 0 | 32 | 21 | 0 |
| *Oriolus oriolus* | 0 | 42 | 9 | 0 | 0 | 62 | 2 | 0 |
| *Turdus philomelos* | 26 | 14 | 5 | 0 | 67 | 27 | 25 | 0 |
| *Carduelis flammea* | 0 | 0 | 4 | 40 | 0 | 0 | 0 | 0 |
| *Turdus merula* | 13 | 18 | 10 | 0 | 17 | 38 | 13 | 4 |
| *Dendrocopos major* | 1 | 11 | 19 | 8 | 1 | 16 | 22 | 6 |
| *Fringilla montifringilla* | 6 | 0 | 30 | 0 | 2 | 0 | 111 | 348 |
| *Haliaeetus albicilla* | 15 | 0 | 4 | 15 | 0 | 2 | 3 | 4 |
| *Phylloscopus trochilus* | 8 | 19 | 4 | 0 | 13 | 18 | 9 | 0 |
| *Emberiza hortulana* | 1 | 29 | 0 | 0 | 4 | 30 | 0 | 0 |
| *Erithacus rubecula* | 4 | 4 | 22 | 0 | 15 | 4 | 25 | 0 |
| *Acrocephalus palustris* | 0 | 28 | 2 | 0 | 0 | 16 | 0 | 0 |
| *Larus argentatus/cachinnans* | 1 | 3 | 2 | 23 | 6 | 0 | 39 | 0 |
| *Phylloscopus collybita* | 7 | 6 | 13 | 0 | 14 | 11 | 16 | 0 |
| *Milvus milvus* | 1 | 10 | 14 | 0 | 4 | 2 | 0 | 0 |
| *Lullula arborea* | 22 | 2 | 1 | 0 | 7 | 6 | 4 | 0 |
| *Cygnus olor* | 13 | 4 | 5 | 2 | 39 | 0 | 45 | 13 |
| *Lanius excubitor* | 2 | 2 | 11 | 9 | 2 | 3 | 10 | 7 |
| *Coturnix coturnix* | 0 | 16 | 6 | 0 | 0 | 33 | 11 | 0 |
| *Apus apus* | 0 | 21 | 1 | 0 | 0 | 35 | 1 | 0 |
| *Sylvia curruca* | 5 | 14 | 1 | 0 | 1 | 11 | 0 | 0 |
| *Buteo lagopus* | 0 | 0 | 3 | 16 | 5 | 0 | 1 | 10 |
| *Accipiter nisus* | 2 | 3 | 10 | 4 | 0 | 2 | 19 | 8 |
| *Anthus trivialis* | 0 | 4 | 15 | 0 | 0 | 4 | 45 | 0 |
| *Larus canus* | 3 | 0 | 15 | 1 | 6 | 0 | 4 | 0 |
| *Luscinia megarhynchos* | 3 | 15 | 0 | 0 | 1 | 9 | 0 | 0 |
| *Pyrrhula pyrrhula* | 2 | 0 | 6 | 9 | 3 | 0 | 9 | 28 |
| *Anser albifrons* | 8 | 0 | 8 | 0 | 28 | 0 | 84 | 60 |
| *Saxicola rubetra* | 3 | 12 | 0 | 0 | 3 | 7 | 6 | 0 |
| *Sylvia borin* | 0 | 14 | 1 | 0 | 0 | 4 | 3 | 0 |
| *Cuculus canorus* | 0 | 12 | 1 | 0 | 1 | 15 | 0 | 0 |
| *Phalacrocorax carbo* | 0 | 2 | 11 | 0 | 1 | 0 | 35 | 23 |
| *Perdix perdix* | 0 | 0 | 0 | 13 | 0 | 2 | 0 | 10 |
| *Serinus serinus* | 3 | 7 | 2 | 0 | 0 | 3 | 7 | 0 |
| *Sitta europaea* | 3 | 0 | 6 | 3 | 2 | 3 | 8 | 3 |
| *Certhia brachydactyla* | 3 | 2 | 6 | 1 | 0 | 0 | 3 | 0 |
| *Accipiter gentilis* | 1 | 2 | 5 | 3 | 1 | 1 | 5 | 4 |
| *Prunella modularis* | 2 | 0 | 5 | 2 | 0 | 0 | 24 | 0 |
| *Anas crecca* | 0 | 9 | 0 | 0 | 0 | 0 | 0 | 0 |
| *Turdus viscivorus* | 0 | 1 | 2 | 4 | 10 | 0 | 7 | 3 |
| *Poecile montanus* | 0 | 1 | 6 | 0 | 1 | 0 | 12 | 2 |
| *Loxia curvirostra* | 6 | 0 | 0 | 0 | 0 | 0 | 0 | 0 |
| *Acrocephalus schoenobaenus* | 1 | 5 | 0 | 0 | 1 | 7 | 0 | 0 |
| *Upupa epops* | 0 | 6 | 0 | 0 | 1 | 6 | 1 | 0 |
| *Turdus iliacus* | 4 | 1 | 0 | 0 | 17 | 0 | 0 | 0 |
| *Fulica atra* | 3 | 2 | 0 | 0 | 10 | 3 | 0 | 0 |
| *Sylvia nisoria* | 0 | 5 | 0 | 0 | 0 | 2 | 0 | 0 |
| *Hippolais icterina* | 0 | 5 | 0 | 0 | 0 | 12 | 0 | 0 |
| *Riparia riparia* | 0 | 5 | 0 | 0 | 0 | 3 | 0 | 0 |
| *Troglodytes troglodytes* | 0 | 0 | 5 | 0 | 1 | 1 | 1 | 0 |
| *Falco columbarius* | 0 | 0 | 3 | 2 | 0 | 0 | 0 | 3 |
| *Acrocephalus arundinaceus* | 0 | 4 | 0 | 0 | 0 | 16 | 0 | 0 |
| *Circus cyaneus* | 0 | 0 | 0 | 4 | 0 | 0 | 0 | 2 |
| *Milvus migrans* | 1 | 2 | 0 | 0 | 0 | 0 | 0 | 0 |
| *Phasianus colchicus* | 1 | 1 | 0 | 1 | 0 | 0 | 2 | 0 |
| *Phoenicurus phoenicurus* | 0 | 3 | 0 | 0 | 0 | 1 | 0 | 0 |
| *Dryocopus martius* | 2 | 0 | 0 | 1 | 1 | 0 | 0 | 1 |
| *Falco tinnunculus* | 0 | 0 | 3 | 0 | 0 | 0 | 1 | 0 |
| *Egretta alba* | 0 | 0 | 3 | 0 | 0 | 0 | 0 | 0 |
| *Aegithalos caudatus* | 0 | 0 | 3 | 0 | 0 | 0 | 0 | 0 |
| *Lophophanes cristatus* | 0 | 0 | 2 | 1 | 0 | 0 | 0 | 0 |
| *Phoenicurus phoenicurus* | 1 | 1 | 0 | 0 | 0 | 1 | 0 | 0 |
| *Numenius arquata* | 1 | 0 | 1 | 0 | 0 | 0 | 5 | 0 |
| *Streptopelia turtur* | 0 | 0 | 2 | 0 | 0 | 2 | 0 | 0 |
| *Circus pygargus* | 0 | 1 | 1 | 0 | 0 | 1 | 0 | 0 |
| *Picus viridis* | 0 | 2 | 0 | 0 | 0 | 3 | 2 | 0 |
| *Falco subbuteo* | 0 | 0 | 2 | 0 | 0 | 4 | 1 | 0 |
| *Larus melanocephalus* | 0 | 0 | 2 | 0 | 0 | 0 | 0 | 0 |
| *Ficedula hypoleuca* | 0 | 0 | 2 | 0 | 0 | 1 | 0 | 0 |
| *Asio otus* | 1 | 0 | 0 | 0 | 0 | 0 | 0 | 0 |
| *Tringa ochropus* | 0 | 1 | 0 | 0 | 1 | 0 | 0 | 0 |
| *Dendrocopos medius* | 0 | 0 | 1 | 0 | 0 | 0 | 0 | 0 |
| *Charadrius dubius* | 0 | 1 | 0 | 0 | 0 | 0 | 0 | 0 |
| *Regulus regulus* | 0 | 1 | 0 | 0 | 0 | 1 | 30 | 6 |
| *Actitis hypoleucos* | 0 | 1 | 0 | 0 | 0 | 0 | 0 | 0 |
| *Muscicapa striata* | 0 | 0 | 1 | 0 | 0 | 0 | 7 | 0 |
| *Pernis apivorus* | 0 | 0 | 1 | 0 | 0 | 1 | 0 | 0 |
| *Locustella naevia* | 0 | 0 | 1 | 0 | 0 | 3 | 0 | 0 |
| *Oenanthe oenanthe* | 0 | 0 | 1 | 0 | 0 | 1 | 0 | 0 |
| *Poecile palustris* | 0 | 0 | 1 | 0 | 0 | 0 | 0 | 0 |
| *Falco sp.* | 0 | 0 | 1 | 0 | 0 | 0 | 0 | 0 |
| *Gallinago gallinago* | 0 | 0 | 1 | 0 | 0 | 0 | 0 | 0 |
| *Cygnus cygnus* | 1 | 0 | 0 | 0 | 1 | 0 | 0 | 10 |
