# Supplementary material for: Constant and seasonal drivers of bird communities in a wind farm: implications for conservation
Source: PeerJ. 2016 Jul 19;4:e2105. doi: 10.7717/peerj.2105 (PMC4957985; doi:10.7717/peerj.2105)
Supplement: Tables S3–S16 — Best models and averaged values of function parameters of variables correlated to Chao 1 bird species richness estimator, abundance of entire bird community, and abundance of five selected farmland species in different seasons and years. [file peerj-04-2105-s003.docx]

**SUPPLEMENTARY MATERIAL**

**Tables S3-S16**. Best models and averaged values of function parameters of variables correlated to Chao 1 bird species richness estimator, abundance of entire bird community, and abundance of five selected farmland species in different seasons and years.

Table S3. Best models describing bird species richness Chao 1 estimator in different seasons and years. For each model the number of explanatory variables (k), log-likelihood, corrected Akaike (AICc), difference between Akaike of a given model and best model (Δ), and model weight (*w*). Explanations of variable abbreviations are given in Table 1. Variable describing the effect of spatial autocorrelation (Auto) were included into each model. Explanations of variable abbreviations are given in Table 1.

| **Model** | **k** | **logLik** | **AICc** | **Δ** | ***w*** |
| --- | --- | --- | --- | --- | --- |
| **Chao 1 estimator** |  |  |  |  |  |
| ***Spring migration 2011*** |  |  |  |  |  |
| Auto+DTurb+Sunrise^2+Sunrise | 4 | -586.75 | 1190.34 | 0 | 0.06 |
| Auto+DTurb+Forest+Sunrise^2+Sunrise | 5 | -586.07 | 1191.2 | 0.86 | 0.04 |
| Auto+Settle+DTurb+Sunrise^2+Sunrise | 5 | -586.43 | 1191.92 | 1.58 | 0.03 |
| Auto+DTurb+Sunrise^2 | 3 | -588.68 | 1192 | 1.66 | 0.03 |
| Auto+DTurb+FieldS+Sunrise^2+Sunrise | 5 | -586.54 | 1192.14 | 1.8 | 0.02 |
| Auto+Settle+DTurb+Forest+Sunrise^2+Sunrise | 6 | -585.51 | 1192.32 | 1.98 | 0.02 |
| ***Spring migration 2012*** |  |  |  |  |  |
| Auto+DTurb+FieldS+Sunrise^2+Sunrise | 5 | -520.04 | 1059.13 | 0 | 0.02 |
| Auto+DTurb+Water+FieldS+Forest+Sunrise^2+Sunrise | 7 | -517.87 | 1059.31 | 0.17 | 0.02 |
| Auto+DTurb+FieldS+Forest+Sunrise^2+Sunrise | 6 | -519.13 | 1059.56 | 0.43 | 0.02 |
| Auto+Settle+DTurb+FieldS+Forest+Sunrise^2+Sunrise | 7 | -518.03 | 1059.62 | 0.49 | 0.02 |
| Auto+Settle+DTurb+Forest+Sunrise^2+Sunrise | 6 | -519.18 | 1059.66 | 0.52 | 0.02 |
| Auto+Settle+DTurb+Water+FieldS+Forest+Sunrise^2+Sunrise | 8 | -516.94 | 1059.74 | 0.61 | 0.02 |
| Auto+Settle+DTurb+FieldS+Sunrise^2+Sunrise | 6 | -519.4 | 1060.1 | 0.97 | 0.01 |
| Auto+Settle+DTurb+Water+Forest+Sunrise^2+Sunrise | 7 | -518.32 | 1060.21 | 1.08 | 0.01 |
| Auto+Settle+DTurb+Forest+Sunrise | 5 | -520.58 | 1060.22 | 1.08 | 0.01 |
| Auto+DTurb+Water+FieldS+Forest+Sunrise | 6 | -519.5 | 1060.31 | 1.17 | 0.01 |
| Auto+Settle+DTurb+Sunrise^2+Sunrise | 5 | -520.74 | 1060.53 | 1.4 | 0.01 |
| Auto+Settle+DTurb+Water+Forest+Sunrise | 6 | -519.63 | 1060.55 | 1.42 | 0.01 |
| Auto+DTurb+FieldS+Sunrise | 4 | -521.86 | 1060.56 | 1.43 | 0.01 |
| Auto+DTurb+Water+FieldS+Sunrise^2+Sunrise | 6 | -519.66 | 1060.63 | 1.5 | 0.01 |
| Auto+DTurb+Water+FieldS+Forest+Sunrise^2+Sunrise+Road | 8 | -517.39 | 1060.65 | 1.51 | 0.01 |
| Auto+DTurb+FieldS+Forest+Sunrise^2+Sunrise+Road | 7 | -518.57 | 1060.72 | 1.58 | 0.01 |
| Auto+DTurb+FieldS+Forest+Sunrise | 5 | -520.85 | 1060.75 | 1.62 | 0.01 |
| Auto+Settle+DTurb+Water+FieldS+Forest+Sunrise | 7 | -518.6 | 1060.76 | 1.63 | 0.01 |
| Auto+DTurb+CV+FieldS+Sunrise^2+Sunrise | 6 | -519.75 | 1060.8 | 1.67 | 0.01 |
| Auto+Settle+DTurb+FieldS+Forest+Sunrise | 6 | -519.77 | 1060.84 | 1.7 | 0.01 |
| Auto+DTurb+FieldS+Sunrise^2+Sunrise+Road | 6 | -519.78 | 1060.86 | 1.72 | 0.01 |
| Auto+Settle+DTurb+CV+Forest+Sunrise | 6 | -519.83 | 1060.97 | 1.83 | 0.01 |
| Auto+DTurb+CV+FieldS+Forest+Sunrise^2+Sunrise | 7 | -518.73 | 1061.04 | 1.9 | 0.01 |
| Auto+Settle+DTurb+CV+FieldS+Forest+Sunrise^2+Sunrise | 8 | -517.59 | 1061.06 | 1.92 | 0.01 |
| Auto+Settle+DTurb+CV+Forest+Sunrise^2+Sunrise | 7 | -518.75 | 1061.08 | 1.94 | 0.01 |
| ***Breeding period 2011*** |  |  |  |  |  |
| Auto+Settle+DTurb+Forest+Sunrise | 5 | -1205.16 | 2428.89 | 0 | 0.02 |
| Auto+Settle+Forest+Sunrise | 4 | -1206.22 | 2428.91 | 0.01 | 0.02 |
| Auto+Settle+Forest+Sunrise^2+Sunrise | 5 | -1205.24 | 2429.06 | 0.17 | 0.02 |
| Auto+Settle+DTurb+Forest+Sunrise^2+Sunrise | 6 | -1204.3 | 2429.3 | 0.4 | 0.02 |
| Auto+Settle+DTurb+Sunrise | 4 | -1206.53 | 2429.51 | 0.62 | 0.01 |
| Auto+DTurb+Sunrise | 3 | -1207.61 | 2429.57 | 0.67 | 0.01 |
| Auto+Settle+FieldS+Forest+Sunrise | 5 | -1205.52 | 2429.62 | 0.73 | 0.01 |
| Auto+Settle+DTurb+Sunrise^2+Sunrise | 5 | -1205.59 | 2429.76 | 0.86 | 0.01 |
| Auto+Settle+DTurb+Forest+Sunrise+NTurb | 6 | -1204.57 | 2429.84 | 0.95 | 0.01 |
| Auto+DTurb+Sunrise^2+Sunrise | 4 | -1206.72 | 2429.91 | 1.01 | 0.01 |
| Auto+DTurb+Forest+Sunrise | 4 | -1206.79 | 2430.03 | 1.14 | 0.01 |
| Auto+Settle+FieldS+Forest+Sunrise^2+Sunrise | 6 | -1204.67 | 2430.05 | 1.16 | 0.01 |
| Auto+DTurb+Forest+Sunrise+Road | 5 | -1205.79 | 2430.15 | 1.26 | 0.01 |
| Auto+Settle+Forest+Sunrise+NTurb | 5 | -1205.82 | 2430.22 | 1.32 | 0.01 |
| Auto+Settle+DTurb+Forest+Sunrise^2+Sunrise+NTurb | 7 | -1203.69 | 2430.23 | 1.33 | 0.01 |
| Auto+Settle+DTurb+Forest+Sunrise+Road | 6 | -1204.77 | 2430.23 | 1.34 | 0.01 |
| Auto+Settle+Forest+Sunrise^2+Sunrise+NTurb | 6 | -1204.82 | 2430.34 | 1.44 | 0.01 |
| Auto+Settle+DTurb+CV+Forest+Sunrise | 6 | -1204.85 | 2430.41 | 1.52 | 0.01 |
| Auto+Settle+CV+Forest+Sunrise^2+Sunrise | 6 | -1204.88 | 2430.47 | 1.57 | 0.01 |
| Auto+Settle+CV+Forest+Sunrise | 5 | -1205.95 | 2430.48 | 1.59 | 0.01 |
| Auto+Settle+DTurb+FieldS+Forest+Sunrise | 6 | -1204.89 | 2430.49 | 1.6 | 0.01 |
| Auto+DTurb+Forest+Sunrise^2+Sunrise | 5 | -1205.97 | 2430.51 | 1.61 | 0.01 |
| Auto+DTurb+Forest+Sunrise^2+Sunrise+Road | 6 | -1204.91 | 2430.52 | 1.62 | 0.01 |
| Auto+Settle+DTurb+Forest+Sunrise^2+Sunrise+Road | 7 | -1203.87 | 2430.58 | 1.68 | 0.01 |
| Auto+Settle+Forest+Sunrise+Road | 5 | -1206.01 | 2430.58 | 1.69 | 0.01 |
| Auto+DTurb+Sunrise+Road | 5 | -1207.06 | 2430.59 | 1.69 | 0.01 |
| Auto+Settle+DTurb+CV+Forest+Sunrise^2+Sunrise | 7 | -1203.9 | 2430.65 | 1.76 | 0.01 |
| Auto+Settle+Forest+Sunrise^2+Sunrise+Road | 6 | -1204.99 | 2430.68 | 1.78 | 0.01 |
| Auto+DTurb+Sunrise^2+Sunrise+Road | 5 | -1206.12 | 2430.81 | 1.92 | 0.01 |
| ***Breeding period 2012*** |  |  |  |  |  |
| Auto+DTurb+FieldS+Forest+Sunrise^2+Road | 6 | -1194.77 | 2410.24 | 0 | 0.04 |
| Auto+Settle+Forest+Sunrise^2 | 4 | -1197.15 | 2410.76 | 0.52 | 0.03 |
| Auto+Settle+DTurb+Forest+Sunrise^2+Road | 6 | -1195.08 | 2410.87 | 0.63 | 0.03 |
| Auto+Settle+DTurb+Forest+Sunrise^2 | 5 | -1196.18 | 2410.93 | 0.69 | 0.03 |
| Auto+Settle+DTurb+FieldS+Forest+Sunrise^2+Road | 7 | -1194.06 | 2410.98 | 0.74 | 0.03 |
| Auto+Settle+Forest+Sunrise^2+Road | 5 | -1196.42 | 2411.4 | 1.16 | 0.02 |
| Auto+Settle+DTurb+FieldS+Forest+Sunrise^2 | 6 | -1195.42 | 2411.54 | 1.3 | 0.02 |
| Auto+DTurb+FieldS+Forest+Sunrise^2+NTurb+Road | 7 | -1194.63 | 2412.1 | 1.86 | 0.02 |
| Auto+DTurb+CV+FieldS+Forest+Sunrise^2+Road | 7 | -1194.67 | 2412.19 | 1.95 | 0.02 |
| ***Autumn migration 2011*** |  |  |  |  |  |
| Auto+Settle+DTurb+Sunrise | 4 | -1410.11 | 2836.5 | 0 | 0.04 |
| Auto+Settle+DTurb+Water+Sunrise | 5 | -1409.52 | 2837.38 | 0.88 | 0.02 |
| Auto+DTurb+FieldS+Sunrise | 4 | -1410.59 | 2837.45 | 0.95 | 0.02 |
| Auto+DTurb+Water+FieldS+Sunrise | 5 | -1409.63 | 2837.6 | 1.1 | 0.02 |
| Auto+Settle+DTurb+FieldS+Sunrise | 5 | -1409.67 | 2837.67 | 1.17 | 0.02 |
| Auto+DTurb+FieldS+Sunrise+Road | 5 | -1409.8 | 2837.95 | 1.45 | 0.02 |
| Auto+Settle+DTurb+Forest+Sunrise | 5 | -1409.83 | 2838 | 1.5 | 0.02 |
| Auto+Settle+DTurb+Sunrise^2+Sunrise | 5 | -1409.84 | 2838.03 | 1.53 | 0.02 |
| Auto+Settle+DTurb+Sunrise+Road | 5 | -1409.85 | 2838.04 | 1.54 | 0.02 |
| Auto+DTurb+Sunrise | 3 | -1411.92 | 2838.05 | 1.55 | 0.02 |
| Auto+Settle+DTurb+Water+FieldS+Sunrise | 6 | -1408.95 | 2838.31 | 1.81 | 0.01 |
| Auto+DTurb+Sunrise+Road | 4 | -1411.02 | 2838.32 | 1.82 | 0.01 |
| Auto+DTurb+FieldS+Forest+Sunrise | 5 | -1410 | 2838.34 | 1.84 | 0.01 |
| Auto+DTurb+Water+Sunrise | 4 | -1411.07 | 2838.42 | 1.92 | 0.01 |
| Auto+Settle+DTurb+Sunrise+NTurb | 5 | -1410.04 | 2838.42 | 1.92 | 0.01 |
| ***Autumn migration 2012*** |  |  |  |  |  |
| Auto+DTurb+Water+FieldS+Sunrise^2 | 5 | -1612.24 | 3242.82 | 0 | 0.04 |
| Auto+DTurb+Water+FieldS+Sunrise^2+Sunrise | 6 | -1611.64 | 3243.69 | 0.87 | 0.02 |
| Auto+DTurb+FieldS+Sunrise^2 | 4 | -1613.72 | 3243.71 | 0.89 | 0.02 |
| Auto+DTurb+Water+FieldS+Sunrise^2+Road | 6 | -1611.68 | 3243.77 | 0.95 | 0.02 |
| Auto+Settle+DTurb+Water+FieldS+Sunrise^2 | 6 | -1611.7 | 3243.81 | 0.99 | 0.02 |
| Auto+Settle+DTurb+FieldS+Sunrise^2 | 5 | -1612.89 | 3244.11 | 1.29 | 0.02 |
| Auto+DTurb+FieldS+Sunrise^2+Road | 5 | -1612.91 | 3244.16 | 1.34 | 0.02 |
| Auto+DTurb+Water+FieldS+Forest+Sunrise^2 | 6 | -1611.95 | 3244.32 | 1.49 | 0.02 |
| Auto+DTurb+FieldS+Sunrise^2+Sunrise | 5 | -1613.14 | 3244.63 | 1.81 | 0.01 |
| Auto+DTurb+Water+FieldS+Sunrise^2+NTurb | 6 | -1612.13 | 3244.68 | 1.86 | 0.01 |
| Auto+DTurb+Water+FieldS+Sunrise^2+Sunrise+Road | 7 | -1611.1 | 3244.7 | 1.88 | 0.01 |
| Auto+Settle+DTurb+Water+FieldS+Sunrise^2+Sunrise | 7 | -1611.12 | 3244.73 | 1.91 | 0.01 |
| ***Winter 2011/2012*** |  |  |  |  |  |
| Auto+DTurb+Sunrise^2 | 3 | -405.67 | 825.88 | 0 | 0.02 |
| Auto+DTurb+Water+Sunrise^2 | 4 | -404.66 | 826.01 | 0.13 | 0.02 |
| Auto+Settle+DTurb+Water+FieldS+Sunrise^2 | 6 | -402.61 | 826.3 | 0.41 | 0.02 |
| Auto+DTurb+Sunrise^2+Sunrise | 4 | -404.81 | 826.31 | 0.42 | 0.02 |
| Auto+DTurb+Water+Sunrise^2+Sunrise | 5 | -403.82 | 826.52 | 0.64 | 0.02 |
| Auto+Settle+DTurb+Water+CV+FieldS+Sunrise^2 | 7 | -401.69 | 826.67 | 0.79 | 0.02 |
| Auto+DTurb+FieldS+Sunrise^2 | 4 | -405.02 | 826.75 | 0.86 | 0.02 |
| Auto+DTurb+Water+FieldS+Sunrise^2 | 5 | -403.96 | 826.79 | 0.91 | 0.02 |
| Auto+Settle+DTurb+Water+FieldS+Sunrise^2+Sunrise | 7 | -401.79 | 826.87 | 0.99 | 0.01 |
| Auto+Settle+DTurb+Water+CV+FieldS+Sunrise^2+Sunrise | 8 | -400.67 | 826.88 | 1 | 0.01 |
| Auto+DTurb+Water+CV+Sunrise^2 | 5 | -404.02 | 826.92 | 1.04 | 0.01 |
| Auto+DTurb+FieldS+Sunrise^2+Sunrise | 5 | -404.11 | 827.09 | 1.21 | 0.01 |
| Auto+DTurb+Water+CV+Sunrise^2+Sunrise | 6 | -403.04 | 827.14 | 1.26 | 0.01 |
| Auto+DTurb+Water+CV+FieldS+Sunrise^2 | 6 | -403.05 | 827.17 | 1.29 | 0.01 |
| Auto+Settle+DTurb+FieldS+Sunrise^2 | 5 | -404.17 | 827.22 | 1.34 | 0.01 |
| Auto+DTurb+Water+CV+FieldS+Sunrise^2+Sunrise | 7 | -401.97 | 827.23 | 1.35 | 0.01 |
| Auto+DTurb+Water+FieldS+Sunrise^2+Sunrise | 6 | -403.08 | 827.24 | 1.35 | 0.01 |
| Auto+Settle+DTurb+Water+Sunrise^2 | 5 | -404.21 | 827.29 | 1.4 | 0.01 |
| Auto+DTurb+Water+Sunrise^2+Road | 5 | -404.25 | 827.37 | 1.49 | 0.01 |
| Auto+DTurb+CV+Sunrise^2 | 4 | -405.39 | 827.48 | 1.6 | 0.01 |
| Auto+DTurb+Sunrise^2+Road | 4 | -405.43 | 827.57 | 1.68 | 0.01 |
| Auto+Settle+DTurb+Sunrise^2 | 4 | -405.44 | 827.57 | 1.68 | 0.01 |
| Auto+Settle+DTurb+FieldS+Sunrise^2+Sunrise | 6 | -403.29 | 827.66 | 1.78 | 0.01 |
| Auto+DTurb+CV+Sunrise^2+Sunrise | 5 | -404.42 | 827.71 | 1.82 | 0.01 |
| Auto+Settle+DTurb+Water+FieldS+Sunrise^2+NTurb | 7 | -402.29 | 827.87 | 1.99 | 0.01 |
| ***Winter 2012/2013*** |  |  |  |  |  |
| Auto+DTurb+Forest+Sunrise+Road | 5 | -451.46 | 921.79 | 0 | 0.02 |
| Auto+DTurb+Forest+Sunrise | 4 | -452.58 | 921.85 | 0.06 | 0.02 |
| Auto+Settle+Forest+Sunrise | 4 | -452.79 | 922.27 | 0.49 | 0.02 |
| Auto+Settle+DTurb+Forest+Sunrise | 5 | -451.92 | 922.72 | 0.93 | 0.02 |
| Auto+DTurb+Forest | 3 | -454.24 | 923.02 | 1.23 | 0.01 |
| Auto+DTurb+Forest+Road | 4 | -453.2 | 923.1 | 1.31 | 0.01 |
| Auto+Forest+Sunrise | 3 | -454.47 | 923.48 | 1.69 | 0.01 |
| Auto+Settle+Forest | 3 | -454.48 | 923.51 | 1.72 | 0.01 |
| Auto+Settle+DTurb+Forest+Sunrise+Road | 6 | -451.23 | 923.53 | 1.75 | 0.01 |
| Auto+Settle+Forest+Sunrise+Road | 5 | -452.38 | 923.63 | 1.84 | 0.01 |
| Auto+Forest+Sunrise+Road | 4 | -453.5 | 923.69 | 1.9 | 0.01 |
| Auto+DTurb+FieldS+Forest+Sunrise | 5 | -452.42 | 923.71 | 1.92 | 0.01 |

Table S4. Averaged values of function parameters of variables that were present in the best models describing Chao 1 estimator of species richness. - Explanations of variable abbreviations are given in Table 1. Intercept – a constant which is the expected mean value of dependent variable when all independent variables equal zero. Statistically significant effects are emboldened.

| **Chao 1 estimator** | Estimate | SE *adj* | -95 % CI | +95 % CI |
| --- | --- | --- | --- | --- |
| *Spring migration 2011* |  |  |  |  |
| **Intercept** | **2.208** | **0.188** | **1.839** | **2.577** |
| Auto | 0.015 | 0.022 | -0.027 | 0.057 |
| CV | 0.030 | 0.073 | -0.114 | 0.174 |
| **DTurb** | **0.285** | **0.085** | **0.118** | **0.452** |
| FieldS | 0.068 | 0.084 | -0.096 | 0.233 |
| Forest | 0.107 | 0.081 | -0.052 | 0.265 |
| NTurb | -0.002 | 0.079 | -0.156 | 0.152 |
| Road | 0.023 | 0.076 | -0.126 | 0.172 |
| Settle | -0.091 | 0.086 | -0.259 | 0.078 |
| **Sunrise** | **-0.067** | **0.034** | **-0.133** | **-0.001** |
| **Sunrise^2** | **-0.173** | **0.039** | **-0.250** | **-0.096** |
| Water | -0.011 | 0.078 | -0.163 | 0.142 |
| *Spring migration 2012* |  |  |  |  |
| **Intercept** | **2.072** | **0.104** | **1.868** | **2.275** |
| **Auto** | **0.148** | **0.037** | **0.075** | **0.221** |
| CV | 0.047 | 0.054 | -0.058 | 0.153 |
| **DTurb** | **0.199** | **0.071** | **0.059** | **0.339** |
| FieldS | -0.106 | 0.060 | -0.224 | 0.013 |
| Forest | 0.114 | 0.064 | -0.011 | 0.239 |
| NTurb | -0.026 | 0.062 | -0.149 | 0.096 |
| Road | 0.034 | 0.057 | -0.078 | 0.146 |
| Settle | -0.109 | 0.069 | -0.244 | 0.026 |
| **Sunrise** | **-0.085** | **0.027** | **-0.138** | **-0.032** |
| Sunrise^2 | 0.054 | 0.031 | -0.007 | 0.115 |
| Water | -0.069 | 0.060 | -0.187 | 0.049 |
| *Breeding period 2011* |  |  |  |  |
| **Intercept** | **2.331** | **0.087** | **2.161** | **2.501** |
| **Auto** | **0.027** | **0.012** | **0.004** | **0.049** |
| CV | -0.049 | 0.066 | -0.179 | 0.080 |
| DTurb | 0.159 | 0.083 | -0.004 | 0.322 |
| FieldS | 0.040 | 0.079 | -0.114 | 0.194 |
| Forest | 0.140 | 0.076 | -0.010 | 0.290 |
| NTurb | 0.051 | 0.072 | -0.089 | 0.191 |
| Road | 0.063 | 0.072 | -0.078 | 0.203 |
| Settle | -0.145 | 0.078 | -0.297 | 0.007 |
| **Sunrise** | **-0.169** | **0.023** | **-0.214** | **-0.124** |
| Sunrise^2 | 0.027 | 0.019 | -0.011 | 0.065 |
| Water | 0.016 | 0.074 | -0.130 | 0.161 |
| *Breeding period 2012* |  |  |  |  |
| **Intercept** | **2.340** | **0.110** | **2.124** | **2.556** |
| **Auto** | **0.100** | **0.029** | **0.044** | **0.157** |
| CV | -0.032 | 0.067 | -0.163 | 0.099 |
| **DTurb** | **0.185** | **0.092** | **0.004** | **0.365** |
| FieldS | -0.114 | 0.079 | -0.269 | 0.040 |
| **Forest** | **0.188** | **0.077** | **0.036** | **0.340** |
| NTurb | 0.017 | 0.075 | -0.130 | 0.165 |
| Road | 0.114 | 0.071 | -0.026 | 0.253 |
| Settle | -0.167 | 0.087 | -0.339 | 0.004 |
| Sunrise | -0.001 | 0.016 | -0.032 | 0.030 |
| **Sunrise^2** | **0.121** | **0.032** | **0.057** | **0.184** |
| Water | 0.009 | 0.076 | -0.139 | 0.158 |
| *Autumn migration 2011* |  |  |  |  |
| **Intercept** | **1.680** | **0.140** | **1.406** | **1.955** |
| **Auto** | **0.049** | **0.013** | **0.024** | **0.074** |
| CV | 0.008 | 0.043 | -0.076 | 0.093 |
| **DTurb** | **0.187** | **0.051** | **0.086** | **0.287** |
| FieldS | -0.061 | 0.047 | -0.154 | 0.031 |
| Forest | -0.036 | 0.049 | -0.132 | 0.060 |
| NTurb | -0.013 | 0.047 | -0.105 | 0.079 |
| Road | 0.043 | 0.045 | -0.045 | 0.131 |
| Settle | -0.073 | 0.050 | -0.170 | 0.025 |
| **Sunrise** | **-0.140** | **0.019** | **-0.177** | **-0.104** |
| Sunrise^2 | -0.019 | 0.028 | -0.074 | 0.036 |
| Water | -0.049 | 0.044 | -0.136 | 0.038 |
| *Autumn migration 2012* |  |  |  |  |
| **Intercept** | **1.918** | **0.143** | **1.638** | **2.198** |
| **Auto** | **0.629** | **0.084** | **0.463** | **0.794** |
| CV | 0.007 | 0.043 | -0.076 | 0.091 |
| **DTurb** | **0.160** | **0.048** | **0.066** | **0.254** |
| **FieldS** | **-0.168** | **0.047** | **-0.260** | **-0.075** |
| Forest | 0.035 | 0.050 | -0.064 | 0.133 |
| NTurb | 0.018 | 0.046 | -0.071 | 0.107 |
| Road | 0.046 | 0.042 | -0.037 | 0.129 |
| Settle | -0.055 | 0.052 | -0.156 | 0.047 |
| Sunrise | 0.018 | 0.017 | -0.015 | 0.050 |
| **Sunrise^2** | **-0.054** | **0.027** | **-0.106** | **-0.001** |
| Water | -0.074 | 0.043 | -0.158 | 0.010 |
| *Winter 2011/2012* |  |  |  |  |
| **Intercept** | **0.884** | **0.149** | **0.592** | **1.177** |
| **Auto** | **0.136** | **0.029** | **0.079** | **0.193** |
| CV | -0.095 | 0.084 | -0.259 | 0.069 |
| **DTurb** | **0.427** | **0.096** | **0.239** | **0.615** |
| FieldS | -0.142 | 0.094 | -0.326 | 0.042 |
| Forest | -0.001 | 0.089 | -0.176 | 0.174 |
| NTurb | 0.031 | 0.085 | -0.137 | 0.198 |
| Road | -0.054 | 0.081 | -0.213 | 0.104 |
| Settle | 0.104 | 0.091 | -0.074 | 0.283 |
| Sunrise | -0.069 | 0.051 | -0.168 | 0.031 |
| **Sunrise^2** | **-0.248** | **0.085** | **-0.413** | **-0.082** |
| Water | -0.131 | 0.079 | -0.285 | 0.024 |
| *Winter 2012/2013* |  |  |  |  |
| **Intercept** | **1.033** | **0.122** | **0.794** | **1.271** |
| **Auto** | **0.359** | **0.063** | **0.235** | **0.482** |
| CV | -0.022 | 0.064 | -0.148 | 0.104 |
| DTurb | 0.119 | 0.075 | -0.028 | 0.267 |
| FieldS | -0.008 | 0.072 | -0.149 | 0.133 |
| **Forest** | **-0.167** | **0.076** | **-0.316** | **-0.019** |
| NTurb | -0.017 | 0.070 | -0.155 | 0.121 |
| Road | 0.087 | 0.067 | -0.044 | 0.219 |
| Settle | -0.093 | 0.072 | -0.235 | 0.049 |
| Sunrise | -0.077 | 0.041 | -0.157 | 0.004 |
| Sunrise^2 | 0.000 | 0.059 | -0.116 | 0.117 |
| Water | -0.028 | 0.073 | -0.170 | 0.115 |

Table S5. Best models describing bird abundance in different seasons and years. For each model the number of explanatory variables (k), log-likelihood, corrected Akaike (AICc), difference between Akaike of a given model and best model (Δ), and model weight (*w*). Explanations of variable abbreviations are given in Table 1. Variable describing the effect of spatial autocorrelation (Auto) were included into each model. Explanations of variable abbreviations are given in Table 1.

| **Model** | **k** | **logLik** | **AICc** | **Δ** | ***w*** |
| --- | --- | --- | --- | --- | --- |
| **Abundance** |  |  |  |  |  |
| ***Spring migration 2011*** |  |  |  |  |  |
| Auto+Settle+DTurb+CV+Sunrise^2+Sunrise | 6 | -2587.31 | 5195.93 | 0 | 0.08 |
| Auto+DTurb+CV+Sunrise^2+Sunrise | 5 | -2589.09 | 5197.24 | 1.31 | 0.04 |
| Auto+Settle+DTurb+CV+Sunrise^2+Sunrise+Road | 7 | -2586.96 | 5197.5 | 1.57 | 0.03 |
| Auto+Settle+DTurb+CV+Sunrise^2+Sunrise+NTurb | 7 | -2586.98 | 5197.54 | 1.61 | 0.03 |
| Auto+Settle+DTurb+CV+Forest+Sunrise^2+Sunrise | 7 | -2587.14 | 5197.84 | 1.91 | 0.03 |
| Auto+CV+Sunrise^2+Sunrise | 4 | -2590.5 | 5197.85 | 1.92 | 0.03 |
| ***Spring migration 2012*** |  |  |  |  |  |
| Auto+Settle+Sunrise^2+Sunrise | 4 | -1551.05 | 3118.94 | 0 | 0.06 |
| Auto+Settle+DTurb+Sunrise^2+Sunrise | 5 | -1550.38 | 3119.82 | 0.89 | 0.04 |
| Auto+DTurb+Sunrise^2+Sunrise | 4 | -1551.55 | 3119.95 | 1.01 | 0.04 |
| Auto+Settle+Water+Sunrise^2+Sunrise | 5 | -1550.93 | 3120.92 | 1.98 | 0.02 |
| ***Breeding period 2011*** |  |  |  |  |  |
| Auto+Settle+DTurb+CV+Sunrise^2+Sunrise | 6 | -3256.24 | 6533.19 | 0 | 0.05 |
| Auto+Settle+DTurb+Water+CV+Sunrise^2+Sunrise | 7 | -3255.36 | 6533.57 | 0.38 | 0.04 |
| Auto+Settle+DTurb+CV+Sunrise^2+Sunrise+Road | 7 | -3255.81 | 6534.48 | 1.29 | 0.02 |
| Auto+Settle+DTurb+Sunrise^2+Sunrise | 6 | -3258.07 | 6534.72 | 1.53 | 0.02 |
| Auto+Settle+DTurb+CV+Sunrise^2+Sunrise+NTurb | 7 | -3256.03 | 6534.9 | 1.71 | 0.02 |
| Auto+Settle+DTurb+CV+Forest+Sunrise^2+Sunrise | 7 | -3256.09 | 6535.03 | 1.84 | 0.02 |
| Auto+Settle+DTurb+Water+CV+Sunrise^2+Sunrise+Road | 8 | -3255.07 | 6535.14 | 1.95 | 0.02 |
| Auto+Settle+DTurb+CV+FieldS+Sunrise^2+Sunrise | 7 | -3256.16 | 6535.17 | 1.98 | 0.02 |
| ***Breeding period 2012*** |  |  |  |  |  |
| Auto+Settle+FieldS+Sunrise+Road | 5 | -1963.95 | 3946.48 | 0 | 0.03 |
| Auto+Settle+FieldS+Sunrise^2+Sunrise+Road | 6 | -1962.99 | 3946.68 | 0.2 | 0.02 |
| Auto+Settle+Sunrise+Road | 4 | -1965.17 | 3946.8 | 0.32 | 0.02 |
| Auto+Settle+FieldS+Sunrise | 4 | -1965.29 | 3947.04 | 0.56 | 0.02 |
| Auto+Settle+Sunrise | 3 | -1966.4 | 3947.16 | 0.68 | 0.02 |
| Auto+Settle+FieldS+Sunrise^2+Sunrise | 5 | -1964.38 | 3947.34 | 0.86 | 0.02 |
| Auto+Settle+DTurb+Sunrise | 4 | -1965.52 | 3947.49 | 1.01 | 0.02 |
| Auto+Settle+Sunrise^2+Sunrise+Road | 5 | -1964.55 | 3947.68 | 1.2 | 0.01 |
| Auto+Settle+DTurb+Sunrise+Road | 5 | -1964.64 | 3947.86 | 1.38 | 0.01 |
| Auto+Settle+Sunrise^2+Sunrise | 4 | -1965.8 | 3948.06 | 1.58 | 0.01 |
| Auto+Settle+CV+FieldS+Sunrise^2+Sunrise+Road | 7 | -1962.63 | 3948.11 | 1.63 | 0.01 |
| Auto+Settle+CV+FieldS+Sunrise+Road | 6 | -1963.71 | 3948.13 | 1.65 | 0.01 |
| Auto+Settle+DTurb+Sunrise^2+Sunrise | 5 | -1964.82 | 3948.21 | 1.73 | 0.01 |
| Auto+FieldS+Sunrise^2+Sunrise+Road | 5 | -1964.84 | 3948.26 | 1.78 | 0.01 |
| Auto+Settle+FieldS+Forest+Sunrise+Road | 6 | -1963.78 | 3948.27 | 1.79 | 0.01 |
| Auto+FieldS+Sunrise+Road | 4 | -1965.92 | 3948.3 | 1.82 | 0.01 |
| Auto+Settle+DTurb+FieldS+Sunrise+Road | 6 | -1963.82 | 3948.34 | 1.86 | 0.01 |
| Auto+Settle+DTurb+FieldS+Sunrise | 5 | -1964.9 | 3948.37 | 1.89 | 0.01 |
| Auto+Settle+CV+Sunrise+Road | 5 | -1964.93 | 3948.44 | 1.95 | 0.01 |
| ***Autumn migration 2011*** |  |  |  |  |  |
| Auto+Water+CV+Sunrise^2+Sunrise | 5 | -12463.53 | 24945.41 | 0 | 0.05 |
| Auto+Water+CV+Sunrise^2+Sunrise+Road | 6 | -12463.01 | 24946.43 | 1.02 | 0.03 |
| Auto+DTurb+Water+CV+Forest+Sunrise^2+Sunrise | 7 | -12461.98 | 24946.47 | 1.06 | 0.03 |
| Auto+Water+CV+Forest+Sunrise^2+Sunrise | 6 | -12463.06 | 24946.53 | 1.13 | 0.03 |
| Auto+Water+CV+Sunrise^2+Sunrise+NTurb | 6 | -12463.08 | 24946.57 | 1.16 | 0.03 |
| Auto+DTurb+Water+CV+Sunrise^2+Sunrise | 6 | -12463.09 | 24946.59 | 1.18 | 0.03 |
| Auto+Settle+Water+CV+Sunrise^2+Sunrise | 6 | -12463.21 | 24946.84 | 1.43 | 0.03 |
| Auto+DTurb+Water+CV+Sunrise^2+Sunrise+NTurb | 7 | -12462.28 | 24947.06 | 1.65 | 0.02 |
| Auto+Water+CV+FieldS+Sunrise^2+Sunrise | 6 | -12463.45 | 24947.32 | 1.91 | 0.02 |
| ***Autumn migration 2012*** |  |  |  |  |  |
| Auto+Settle+Water+Sunrise^2+Sunrise+NTurb | 6 | -12256.62 | 24533.65 | 0 | 0.04 |
| Auto+Settle+DTurb+Water+Sunrise^2+Sunrise+NTurb | 7 | -12255.78 | 24534.06 | 0.41 | 0.04 |
| Auto+Settle+DTurb+Sunrise^2+Sunrise+NTurb | 6 | -12257.29 | 24534.99 | 1.35 | 0.02 |
| Auto+Settle+Water+CV+Sunrise^2+Sunrise+NTurb | 7 | -12256.29 | 24535.07 | 1.43 | 0.02 |
| Auto+Settle+DTurb+Water+Sunrise^2+Sunrise | 6 | -12257.35 | 24535.11 | 1.47 | 0.02 |
| Auto+Settle+Water+Sunrise^2+Sunrise+NTurb+Road | 7 | -12256.43 | 24535.37 | 1.72 | 0.02 |
| Auto+Settle+DTurb+Sunrise^2+Sunrise | 5 | -12258.52 | 24535.37 | 1.73 | 0.02 |
| Auto+Settle+Water+Forest+Sunrise^2+Sunrise+NTurb | 7 | -12256.45 | 24535.4 | 1.76 | 0.02 |
| Auto+Settle+DTurb+Water+CV+Sunrise^2+Sunrise+NTurb | 8 | -12255.47 | 24535.54 | 1.89 | 0.02 |
| Auto+Settle+Sunrise^2+Sunrise+NTurb | 5 | -12258.63 | 24535.59 | 1.95 | 0.02 |
| ***Winter 2011/2012*** |  |  |  |  |  |
| Auto+DTurb+CV+FieldS+Sunrise | 5 | -1193.92 | 2406.71 | 0 | 0.05 |
| Auto+DTurb+CV+FieldS+Sunrise+NTurb | 6 | -1193.28 | 2407.64 | 0.93 | 0.03 |
| Auto+DTurb+CV+FieldS+Forest+Sunrise | 6 | -1193.38 | 2407.83 | 1.12 | 0.03 |
| Auto+DTurb+Water+CV+FieldS+Sunrise | 6 | -1193.45 | 2407.98 | 1.27 | 0.03 |
| Auto+DTurb+CV+FieldS+Sunrise^2+Sunrise | 6 | -1193.71 | 2408.48 | 1.77 | 0.02 |
| Auto+CV+FieldS+Sunrise | 4 | -1195.95 | 2408.59 | 1.87 | 0.02 |
| ***Winter 2012/2013*** |  |  |  |  |  |
| Auto+Water+CV+Sunrise^2+Sunrise+NTurb | 6 | -2100.51 | 4222.09 | 0 | 0.04 |
| Auto+CV+FieldS+Forest+Sunrise^2+Sunrise+NTurb+Road | 8 | -2098.36 | 4222.25 | 0.16 | 0.03 |
| Auto+CV+FieldS+Forest+Sunrise^2+Sunrise+NTurb | 7 | -2099.54 | 4222.37 | 0.27 | 0.03 |
| Auto+Water+CV+FieldS+Sunrise^2+Sunrise+NTurb | 7 | -2099.55 | 4222.39 | 0.3 | 0.03 |
| Auto+Water+CV+FieldS+Sunrise^2+Sunrise+NTurb+Road | 8 | -2098.49 | 4222.53 | 0.43 | 0.03 |
| Auto+CV+FieldS+Sunrise^2+Sunrise+NTurb+Road | 7 | -2099.77 | 4222.83 | 0.74 | 0.02 |
| Auto+Water+CV+Sunrise^2+Sunrise+NTurb+Road | 7 | -2099.78 | 4222.86 | 0.77 | 0.02 |
| Auto+CV+Forest+Sunrise^2+Sunrise+NTurb | 6 | -2100.9 | 4222.87 | 0.78 | 0.02 |
| Auto+CV+Sunrise^2+Sunrise+NTurb+Road | 6 | -2100.99 | 4223.05 | 0.95 | 0.02 |
| Auto+CV+Sunrise^2+Sunrise+NTurb | 5 | -2102.09 | 4223.05 | 0.96 | 0.02 |
| Auto+Water+CV+FieldS+Forest+Sunrise^2+Sunrise+NTurb | 8 | -2098.85 | 4223.23 | 1.14 | 0.02 |
| Auto+CV+Forest+Sunrise^2+Sunrise+NTurb+Road | 7 | -2100.08 | 4223.46 | 1.37 | 0.02 |
| Auto+Water+CV+FieldS+Forest+Sunrise^2+Sunrise+NTurb+Road | 9 | -2097.87 | 4223.53 | 1.44 | 0.02 |
| Auto+Water+CV+Forest+Sunrise^2+Sunrise+NTurb | 7 | -2100.13 | 4223.56 | 1.47 | 0.02 |
| Auto+CV+FieldS+Sunrise^2+Sunrise+NTurb | 6 | -2101.26 | 4223.6 | 1.51 | 0.02 |
| Auto+CV+FieldS+Forest+Sunrise^2+Sunrise+Road | 7 | -2100.21 | 4223.72 | 1.62 | 0.02 |

Table S6. Averaged values of function parameters of variables that were present in the best models describing bird abundance. Explanations of variable abbreviations are given in Table 1. Intercept – a constant which is the expected mean value of dependent variable when all independent variables equal zero. Statistically significant effects are emboldened.

| **Abundance** | Estimate | SE *adj* | -95 % CI | +95 % CI |
| --- | --- | --- | --- | --- |
| *Spring migration 2011* |  |  |  |  |
| **Intercept** | **3.389** | **0.343** | **2.717** | **4.061** |
| **Auto** | **0.111** | **0.015** | **0.082** | **0.141** |
| **CV** | **0.250** | **0.114** | **0.027** | **0.474** |
| **DTurb** | **0.262** | **0.133** | **0.001** | **0.522** |
| FieldS | 0.059 | 0.136 | -0.207 | 0.325 |
| Forest | -0.024 | 0.136 | -0.290 | 0.242 |
| NTurb | 0.062 | 0.129 | -0.192 | 0.316 |
| Road | 0.049 | 0.127 | -0.199 | 0.297 |
| Settle | 0.217 | 0.132 | -0.041 | 0.475 |
| **Sunrise** | **-0.126** | **0.024** | **-0.172** | **-0.079** |
| **Sunrise^2** | **-0.423** | **0.023** | **-0.468** | **-0.379** |
| Water | 0.004 | 0.123 | -0.237 | 0.245 |
| *Spring migration 2012* |  |  |  |  |
| **Intercept** | **2.898** | **0.248** | **2.411** | **3.384** |
| **Auto** | **0.414** | **0.068** | **0.282** | **0.547** |
| CV | 0.030 | 0.092 | -0.151 | 0.210 |
| DTurb | 0.155 | 0.104 | -0.049 | 0.358 |
| FieldS | -0.037 | 0.105 | -0.242 | 0.168 |
| Forest | -0.036 | 0.105 | -0.243 | 0.170 |
| NTurb | 0.030 | 0.097 | -0.160 | 0.220 |
| Road | 0.016 | 0.100 | -0.179 | 0.211 |
| Settle | -0.173 | 0.099 | -0.366 | 0.021 |
| **Sunrise** | **-0.326** | **0.017** | **-0.358** | **-0.293** |
| **Sunrise^2** | **0.075** | **0.021** | **0.033** | **0.117** |
| Water | 0.032 | 0.095 | -0.155 | 0.219 |
| *Breeding period 2011* |  |  |  |  |
| **Intercept** | **2.998** | **0.266** | **2.476** | **3.519** |
| **Auto** | **-0.227** | **0.015** | **-0.257** | **-0.197** |
| **CV** | **-0.159** | **0.079** | **-0.313** | **-0.004** |
| DTurb | -0.180 | 0.094 | -0.363 | 0.004 |
| FieldS | -0.083 | 0.094 | -0.267 | 0.100 |
| Forest | -0.059 | 0.095 | -0.245 | 0.127 |
| NTurb | -0.036 | 0.090 | -0.213 | 0.141 |
| Road | 0.103 | 0.087 | -0.067 | 0.274 |
| **Settle** | **-0.192** | **0.094** | **-0.377** | **-0.007** |
| **Sunrise** | **0.038** | **0.017** | **0.005** | **0.071** |
| **Sunrise^2** | **0.047** | **0.011** | **0.025** | **0.070** |
| Water | -0.103 | 0.084 | -0.267 | 0.061 |
| *Breeding period 2012* |  |  |  |  |
| **Intercept** | **3.083** | **0.122** | **2.845** | **3.322** |
| **Auto** | **0.241** | **0.042** | **0.159** | **0.324** |
| CV | -0.047 | 0.060 | -0.165 | 0.071 |
| DTurb | -0.068 | 0.076 | -0.218 | 0.082 |
| FieldS | -0.107 | 0.067 | -0.239 | 0.025 |
| Forest | 0.032 | 0.066 | -0.098 | 0.163 |
| NTurb | -0.009 | 0.066 | -0.138 | 0.120 |
| Road | 0.107 | 0.064 | -0.020 | 0.233 |
| **Settle** | **-0.169** | **0.072** | **-0.310** | **-0.027** |
| **Sunrise** | **0.050** | **0.013** | **0.025** | **0.076** |
| Sunrise^2 | 0.034 | 0.026 | -0.017 | 0.085 |
| Water | 0.011 | 0.063 | -0.111 | 0.134 |
| *Autumn migration 2011* |  |  |  |  |
| **Intercept** | **3.047** | **0.263** | **2.533** | **3.562** |
| **Auto** | **-0.054** | **0.008** | **-0.069** | **-0.039** |
| **CV** | **-0.161** | **0.071** | **-0.299** | **-0.022** |
| DTurb | 0.089 | 0.077 | -0.061 | 0.239 |
| FieldS | 0.027 | 0.073 | -0.116 | 0.170 |
| Forest | -0.102 | 0.086 | -0.270 | 0.066 |
| NTurb | 0.076 | 0.078 | -0.077 | 0.229 |
| Road | 0.068 | 0.072 | -0.072 | 0.208 |
| Settle | -0.049 | 0.075 | -0.196 | 0.099 |
| **Sunrise** | **-0.154** | **0.007** | **-0.167** | **-0.140** |
| **Sunrise^2** | **0.270** | **0.011** | **0.248** | **0.292** |
| **Water** | **-0.174** | **0.075** | **-0.320** | **-0.027** |
| *Autumn migration 2012* |  |  |  |  |
| **Intercept** | **3.234** | **0.186** | **2.869** | **3.600** |
| **Auto** | **2.310** | **0.036** | **2.240** | **2.381** |
| CV | 0.059 | 0.086 | -0.109 | 0.228 |
| DTurb | -0.143 | 0.098 | -0.335 | 0.049 |
| FieldS | 0.063 | 0.098 | -0.129 | 0.254 |
| Forest | -0.087 | 0.100 | -0.282 | 0.109 |
| NTurb | 0.146 | 0.087 | -0.024 | 0.316 |
| Road | 0.069 | 0.091 | -0.109 | 0.247 |
| **Settle** | **-0.254** | **0.095** | **-0.440** | **-0.068** |
| **Sunrise** | **-0.068** | **0.007** | **-0.082** | **-0.053** |
| **Sunrise^2** | **0.036** | **0.013** | **0.010** | **0.062** |
| Water | -0.134 | 0.085 | -0.301 | 0.032 |
| *Winter 2011/2012* |  |  |  |  |
| **Intercept** | **1.609** | **0.209** | **1.200** | **2.018** |
| **Auto** | **-0.807** | **0.040** | **-0.885** | **-0.730** |
| **CV** | **-0.345** | **0.153** | **-0.646** | **-0.045** |
| **DTurb** | **0.344** | **0.172** | **0.005** | **0.682** |
| **FieldS** | **-0.364** | **0.165** | **-0.688** | **-0.040** |
| Forest | -0.103 | 0.175 | -0.447 | 0.240 |
| NTurb | 0.166 | 0.183 | -0.193 | 0.525 |
| Road | 0.027 | 0.157 | -0.280 | 0.334 |
| Settle | -0.084 | 0.202 | -0.480 | 0.312 |
| **Sunrise** | **-0.097** | **0.027** | **-0.150** | **-0.044** |
| Sunrise^2 | -0.043 | 0.064 | -0.169 | 0.082 |
| Water | -0.113 | 0.160 | -0.426 | 0.200 |
| *Winter 2012/2013* |  |  |  |  |
| **Intercept** | **2.443** | **0.301** | **1.853** | **3.033** |
| **Auto** | **2.248** | **0.101** | **2.051** | **2.445** |
| **CV** | **0.314** | **0.136** | **0.047** | **0.581** |
| DTurb | -0.074 | 0.150 | -0.369 | 0.220 |
| FieldS | 0.236 | 0.139 | -0.036 | 0.508 |
| Forest | -0.229 | 0.156 | -0.534 | 0.077 |
| **NTurb** | **0.345** | **0.151** | **0.049** | **0.640** |
| Road | 0.196 | 0.133 | -0.064 | 0.456 |
| Settle | 0.043 | 0.147 | -0.245 | 0.331 |
| **Sunrise** | **-0.097** | **0.017** | **-0.130** | **-0.064** |
| **Sunrise^2** | **-0.263** | **0.028** | **-0.318** | **-0.208** |
| Water | -0.199 | 0.141 | -0.476 | 0.079 |

Table S7. Best models describing abundance of skylark *Alauda arvensis* in different seasons and years. For each model number of explanatory variables (k), log-likelihood, corrected Akaike (AICc), difference between Akaike of a given model and best model (Δ), and model weight (*w*). Explanations of variable abbreviations are given in Table 1. Variable describing the effect of spatial autocorrelation (Auto) were included into each model. Explanations of variable abbreviations are given in Table 1.

| **Model** | **k** | **logLik** | **AICc** | **delta** | **weight** |
| --- | --- | --- | --- | --- | --- |
| ***Alauda arvensis*** |  |  |  |  |  |
| ***Spring migration 2011*** |  |  |  |  |  |
| Auto+Settle+Water+NTurb | 4 | -356.31 | 729.47 | 0.00 | 0.45 |
| Auto+Settle+Water+CV+NTurb | 5 | -355.93 | 730.93 | 1.46 | 0.22 |
| Auto+Settle+Water+Sunrise+NTurb | 5 | -356.16 | 731.38 | 1.92 | 0.17 |
| Auto+Settle+Water+Forest+NTurb | 5 | -356.19 | 731.44 | 1.97 | 0.17 |
| ***Spring migration 2012*** |  |  |  |  |  |
| Auto+Settle+Water+Forest+Sunrise+Road | 6 | -361.50 | 744.31 | 0.00 | 0.37 |
| Auto+Settle+Water+Forest+Road | 5 | -363.20 | 745.47 | 1.15 | 0.21 |
| Auto+Settle+Water+Forest+Sunrise+Sunrise^2+Road | 7 | -361.26 | 746.09 | 1.78 | 0.15 |
| Auto+Settle+DTurb+Water+Forest+Sunrise+Road | 7 | -361.30 | 746.17 | 1.86 | 0.14 |
| Auto+Settle+Water+FieldS+Forest+Sunrise+Road | 7 | -361.36 | 746.29 | 1.98 | 0.14 |
| ***Breeding period 2011*** |  |  |  |  |  |
| Auto+Settle+Water+Forest | 4 | -675.16 | 1366.78 | 0.00 | 0.20 |
| Auto+Settle+Forest | 3 | -676.67 | 1367.70 | 0.92 | 0.13 |
| Auto+Settle+DTurb+Water+Forest | 5 | -674.57 | 1367.70 | 0.92 | 0.13 |
| Auto+Settle+DTurb+Water | 4 | -675.81 | 1368.08 | 1.30 | 0.11 |
| Auto+Settle+Water+Forest+Sunrise^2 | 5 | -674.87 | 1368.32 | 1.54 | 0.09 |
| Auto+Settle+Water+CV+Forest | 5 | -674.89 | 1368.36 | 1.58 | 0.09 |
| Auto+Settle+Water+Forest+Sunrise | 5 | -674.91 | 1368.39 | 1.61 | 0.09 |
| Auto+Settle+Water+Forest+NTurb | 5 | -674.99 | 1368.56 | 1.78 | 0.08 |
| Auto+Settle+DTurb+Forest | 4 | -676.13 | 1368.72 | 1.94 | 0.08 |
| ***Breeding period 2012*** |  |  |  |  |  |
| Auto+Settle+Water+Forest | 4 | -595.67 | 1207.79 | 0.00 | 0.15 |
| Auto+Settle+Water+Forest+Road | 5 | -594.71 | 1207.99 | 0.20 | 0.14 |
| Auto+DTurb+Water+Forest+Road | 5 | -594.75 | 1208.08 | 0.28 | 0.13 |
| Auto+Settle+DTurb+Water+Forest+Road | 6 | -594.01 | 1208.72 | 0.92 | 0.10 |
| Auto+Settle+DTurb+Water+Forest | 5 | -595.22 | 1209.01 | 1.22 | 0.08 |
| Auto+Water+Forest+Road | 4 | -596.33 | 1209.13 | 1.33 | 0.08 |
| Auto+DTurb+Water+Road | 4 | -596.34 | 1209.13 | 1.34 | 0.08 |
| Auto+Settle+Water+Forest+Sunrise | 5 | -595.53 | 1209.63 | 1.84 | 0.06 |
| Auto+Settle+Water+FieldS+Forest | 5 | -595.56 | 1209.69 | 1.89 | 0.06 |
| Auto+Settle+Water+Forest+Sunrise+Road | 6 | -594.52 | 1209.74 | 1.94 | 0.06 |
| Auto+Settle+DTurb+Water | 4 | -596.67 | 1209.79 | 1.99 | 0.06 |
| ***Autumn migration 2011*** |  |  |  |  |  |
| Auto+Forest+Sunrise+Sunrise^2 | 4 | -541.57 | 1099.55 | 0.00 | 0.28 |
| Auto+FieldS+Forest+Sunrise+Sunrise^2 | 5 | -540.83 | 1100.18 | 0.62 | 0.21 |
| Auto+Forest+Sunrise+Sunrise^2+Road | 5 | -541.20 | 1100.92 | 1.37 | 0.14 |
| Auto+Dturb+Forest+Sunrise+Sunrise^2 | 5 | -541.27 | 1101.06 | 1.51 | 0.13 |
| Auto+FieldS+Forest+Sunrise+Sunrise^2+Road | 6 | -540.32 | 1101.27 | 1.72 | 0.12 |
| Auto+Dturb+FieldS+Forest+Sunrise+Sunrise^2 | 6 | -540.35 | 1101.33 | 1.77 | 0.12 |
| ***Autumn migration 2012*** |  |  |  |  |  |
| Auto+Settle+Sunrise+Sunrise^2 | 4 | -333.92 | 684.30 | 0.00 | 0.13 |
| Auto+Settle+Sunrise+Sunrise^2+NTurb | 5 | -333.05 | 684.67 | 0.37 | 0.11 |
| Auto+Sunrise+Sunrise^2 | 3 | -335.23 | 684.81 | 0.51 | 0.10 |
| Auto+Sunrise+Sunrise^2+Road | 4 | -334.32 | 685.10 | 0.80 | 0.09 |
| Auto+Settle+Sunrise+Sunrise^2+Road | 5 | -333.48 | 685.54 | 1.24 | 0.07 |
| Auto+Sunrise+Sunrise^2+NTurb | 4 | -334.55 | 685.55 | 1.25 | 0.07 |
| Auto+Settle+Forest+Sunrise+Sunrise^2+NTurb | 6 | -332.45 | 685.61 | 1.31 | 0.07 |
| Auto+Settle+Water+Sunrise+Sunrise^2 | 5 | -333.56 | 685.69 | 1.39 | 0.06 |
| Auto+Settle+Water+Sunrise+Sunrise^2+NTurb | 6 | -332.68 | 686.06 | 1.76 | 0.05 |
| Auto+Water+Sunrise+Sunrise^2+Road | 5 | -333.78 | 686.12 | 1.82 | 0.05 |
| Auto+Settle+Forest+Sunrise+Sunrise^2 | 5 | -333.78 | 686.13 | 1.83 | 0.05 |
| Auto+Sunrise+Sunrise^2+NTurb+Road | 5 | -333.82 | 686.21 | 1.91 | 0.05 |
| Auto+Settle+Sunrise+Sunrise^2+NTurb+Road | 6 | -332.78 | 686.26 | 1.96 | 0.05 |
| Auto+Settle+CV+Sunrise+Sunrise^2 | 5 | -333.85 | 686.27 | 1.97 | 0.05 |

Table S8. Averaged values of function parameters of variables that were present in the best models describing abundance of skylark *Alauda arvensis*. Explanations of variable abbreviations are given in Table 1. Intercept – a constant which is the expected mean value of dependent variable when all independent variables equal zero. Statistically significant effects are emboldened.

| ***Alauda arvensis*** | Estimate | SE *adj* | -95 % CI | +95 % CI |
| --- | --- | --- | --- | --- |
| *Spring migration 2011* |  |  |  |  |
| **Intercept** | **1.458** | **0.041** | **1.378** | **1.538** |
| **Settle** | **0.155** | **0.041** | **0.075** | **0.235** |
| **Water** | **-0.143** | **0.042** | **-0.226** | **-0.060** |
| **NTurb** | **0.117** | **0.041** | **0.037** | **0.197** |
| **Auto** | **0.433** | **0.176** | **0.089** | **0.777** |
| CV | -0.036 | 0.041 | -0.117 | 0.045 |
| Sunrise | 0.021 | 0.038 | -0.054 | 0.096 |
| Forest | -0.024 | 0.049 | -0.121 | 0.072 |
| *Spring migration 2012* |  |  |  |  |
| **Intercept** | **1.465** | **0.061** | **1.345** | **1.585** |
| **Settle** | **0.085** | **0.039** | **0.009** | **0.161** |
| **Water** | **-0.096** | **0.041** | **-0.175** | **-0.016** |
| **Forest** | **-0.083** | **0.042** | **-0.165** | **-0.001** |
| Sunrise | 0.068 | 0.038 | -0.006 | 0.142 |
| **Road** | **-0.094** | **0.041** | **-0.173** | **-0.014** |
| **Auto** | **0.421** | **0.083** | **0.259** | **0.584** |
| Sunrise^2 | 0.024 | 0.035 | -0.045 | 0.093 |
| DTurb | -0.026 | 0.042 | -0.108 | 0.055 |
| FieldS | -0.021 | 0.039 | -0.096 | 0.055 |
| *Breeding period 2011* |  |  |  |  |
| **Intercept** | **1.356** | **0.126** | **1.108** | **1.603** |
| **Settle** | **0.181** | **0.050** | **0.084** | **0.278** |
| Water | -0.095 | 0.052 | -0.197 | 0.007 |
| **Forest** | **-0.120** | **0.056** | **-0.229** | **-0.011** |
| **Auto** | **0.615** | **0.080** | **0.458** | **0.771** |
| DTurb | -0.070 | 0.055 | -0.177 | 0.038 |
| Sunrise^2 | -0.021 | 0.028 | -0.076 | 0.034 |
| CV | -0.034 | 0.046 | -0.124 | 0.057 |
| Sunrise | 0.026 | 0.037 | -0.046 | 0.099 |
| NTurb | 0.028 | 0.049 | -0.067 | 0.124 |
| *Breeding period 2012* |  |  |  |  |
| **Intercept** | **1.060** | **0.235** | **0.600** | **1.521** |
| Settle | 0.066 | 0.037 | -0.007 | 0.138 |
| **Water** | **-0.099** | **0.038** | **-0.173** | **-0.025** |
| **Forest** | **-0.086** | **0.040** | **-0.165** | **-0.007** |
| **Auto** | **0.580** | **0.109** | **0.367** | **0.793** |
| Road | -0.060 | 0.037 | -0.132 | 0.012 |
| DTurb | -0.059 | 0.040 | -0.137 | 0.020 |
| Sunrise | -0.018 | 0.031 | -0.079 | 0.043 |
| FieldS | -0.017 | 0.035 | -0.086 | 0.052 |
| *Autumn migration 2011* |  |  |  |  |
| Intercept | -1.831 | 1.020 | -3.831 | 0.169 |
| **Forest** | **-0.645** | **0.165** | **-0.969** | **-0.322** |
| **Sunrise** | **0.555** | **0.074** | **0.411** | **0.699** |
| **Sunrise^2** | **-0.450** | **0.107** | **-0.661** | **-0.240** |
| **Auto** | **2.924** | **0.310** | **2.317** | **3.532** |
| FieldS | 0.197 | 0.152 | -0.100 | 0.495 |
| Road | 0.141 | 0.152 | -0.156 | 0.439 |
| DTurb | -0.137 | 0.157 | -0.444 | 0.170 |
| *Autumn migration 2012* |  |  |  |  |
| Intercept | -0.574 | 0.578 | -1.707 | 0.560 |
| Settle | 0.236 | 0.144 | -0.046 | 0.518 |
| **Sunrise** | **0.596** | **0.100** | **0.399** | **0.793** |
| **Sunrise^2** | **-0.858** | **0.123** | **-1.099** | **-0.617** |
| **Auto** | **1.698** | **0.367** | **0.978** | **2.418** |
| NTurb | -0.197 | 0.153 | -0.496 | 0.102 |
| Road | -0.174 | 0.152 | -0.472 | 0.124 |
| Forest | -0.121 | 0.145 | -0.406 | 0.164 |
| Water | -0.132 | 0.143 | -0.411 | 0.147 |
| CV | 0.058 | 0.149 | -0.234 | 0.350 |

Table S9. Best models describing abundance of yellowhammer *Emberiza citrinella* in different seasons and years. For each model the number of explanatory variables (k), log-likelihood, corrected Akaike (AICc), difference between Akaike of a given model and best model (Δ), and model weight (*w*). Explanations of variable abbreviations are given in Table 1. Variable describing the effect of spatial autocorrelation (Auto) were included into each model. Explanations of variable abbreviations are given in Table 1.

| **Model** | **k** | **logLik** | **AICc** | **delta** | **weight** |
| --- | --- | --- | --- | --- | --- |
| ***Emberiza citrinella*** |  |  |  |  |  |
| ***Spring migration 2011*** |  |  |  |  |  |
| Auto+Water+CV+Forest | 4 | -143.88 | 304.61 | 0.00 | 0.19 |
| Auto+Water+CV+Forest+NTurb | 5 | -142.96 | 304.98 | 0.38 | 0.16 |
| Auto+CV+Forest | 3 | -145.32 | 305.29 | 0.68 | 0.13 |
| Auto+Settle+Water+CV+Forest | 5 | -143.30 | 305.66 | 1.05 | 0.11 |
| Auto+Settle+Water+CV+Forest+NTurb | 6 | -142.25 | 305.81 | 1.20 | 0.10 |
| Auto+Settle+CV+Forest | 4 | -144.63 | 306.10 | 1.49 | 0.09 |
| Auto+CV+Forest+NTurb | 4 | -144.83 | 306.49 | 1.89 | 0.07 |
| Auto+Water+CV+FieldS+Forest | 5 | -143.77 | 306.60 | 1.99 | 0.07 |
| Auto+Water+CV+Forest+Road | 5 | -143.77 | 306.60 | 1.99 | 0.07 |
| ***Spring migration 2012*** |  |  |  |  |  |
| Auto+DTurb+Road | 3 | -184.64 | 383.93 | 0.00 | 0.26 |
| Auto+DTurb+Forest+Road | 4 | -184.04 | 384.92 | 0.99 | 0.16 |
| Auto+DTurb+Sunrise+Road | 4 | -184.17 | 385.18 | 1.25 | 0.14 |
| Auto+Settle+DTurb+Road | 4 | -184.26 | 385.36 | 1.43 | 0.13 |
| Auto+DTurb+CV+Road | 4 | -184.39 | 385.62 | 1.68 | 0.11 |
| Auto+DTurb+NTurb+Road | 4 | -184.39 | 385.63 | 1.69 | 0.11 |
| Auto+DTurb+Water+Road | 4 | -184.47 | 385.79 | 1.85 | 0.10 |
| ***Breeding period 2011*** |  |  |  |  |  |
| Auto+DTurb+CV+Forest | 4 | -254.09 | 524.64 | 0.00 | 0.07 |
| Auto+DTurb+CV+Forest+NTurb | 5 | -253.06 | 524.68 | 0.05 | 0.07 |
| Auto+DTurb+Forest | 3 | -255.19 | 524.73 | 0.10 | 0.07 |
| Auto+DTurb+Forest+Sunrise^2 | 4 | -254.14 | 524.74 | 0.11 | 0.07 |
| Auto+DTurb+CV+Forest+Sunrise^2 | 5 | -253.22 | 525.01 | 0.37 | 0.06 |
| Auto+CV+Forest | 3 | -255.37 | 525.09 | 0.46 | 0.06 |
| Auto+Forest | 2 | -256.48 | 525.23 | 0.60 | 0.05 |
| Auto+Forest+Sunrise^2 | 3 | -255.53 | 525.41 | 0.77 | 0.05 |
| Auto+CV+Forest+Sunrise^2 | 4 | -254.57 | 525.60 | 0.97 | 0.04 |
| Auto+DTurb+CV+Forest+Sunrise^2+NTurb | 6 | -252.46 | 525.62 | 0.98 | 0.04 |
| Auto+DTurb+Water+CV+Forest+NTurb | 60 | -252.57 | 525.83 | 1.20 | 0.04 |
| Auto+DTurb+Water+Forest+Sunrise^2 | 5 | -253.71 | 525.99 | 1.35 | 0.04 |
| Auto+DTurb+Water+Forest | 4 | -254.77 | 526.00 | 1.36 | 0.04 |
| Auto+DTurb+Forest+Sunrise+Sunrise^2 | 5 | -253.73 | 526.03 | 1.40 | 0.03 |
| Auto+CV+Forest+NTurb | 4 | -254.89 | 526.23 | 1.59 | 0.03 |
| Auto+DTurb+Water+CV+Forest | 5 | -253.86 | 526.29 | 1.65 | 0.03 |
| Auto+Forest+Sunrise+Sunrise^2 | 4 | -254.93 | 526.33 | 1.69 | 0.03 |
| Auto+DTurb+Forest+NTurb | 4 | -254.96 | 526.37 | 1.74 | 0.03 |
| Auto+Settle+CV+Forest | 4 | -255.02 | 526.49 | 1.85 | 0.03 |
| Auto+Water+Forest | 3 | -256.07 | 526.49 | 1.86 | 0.03 |
| Auto+Settle+Forest+Sunrise^2 | 4 | -255.02 | 526.51 | 1.87 | 0.03 |
| Auto+DTurb+CV+Forest+Road | 5 | -253.98 | 526.54 | 1.90 | 0.03 |
| Auto+DTurb+Forest+Sunrise^2+NTurb | 5 | -254.00 | 526.58 | 1.94 | 0.03 |
| Auto+DTurb+Water+CV+Forest+Sunrise^2 | 6 | -252.97 | 526.64 | 2.00 | 0.03 |
| ***Breeding period 2012*** |  |  |  |  |  |
| Auto+DTurb+Water+CV+Forest+Sunrise+NTurb | 7 | -349.46 | 721.77 | 0.00 | 0.09 |
| Auto+Settle+FieldS+Forest+Sunrise | 5 | -351.67 | 721.92 | 0.15 | 0.08 |
| Auto+DTurb+Water+Forest+Sunrise+NTurb | 6 | -350.65 | 722.01 | 0.23 | 0.08 |
| Auto+Settle+CV+FieldS+Forest+Sunrise | 6 | -350.92 | 722.55 | 0.77 | 0.06 |
| Auto+Settle+Water+FieldS+Forest+Sunrise | 6 | -350.94 | 722.58 | 0.81 | 0.06 |
| Auto+DTurb+CV+Forest+Sunrise+NTurb | 6 | -350.97 | 722.65 | 0.88 | 0.06 |
| Auto+DTurb+Water+Forest+Sunrise | 5 | -352.24 | 723.05 | 1.27 | 0.05 |
| Auto+Settle+CV+FieldS+Forest+Sunrise+NTurb | 7 | -350.16 | 723.17 | 1.40 | 0.04 |
| Auto+DTurb+Forest+Sunrise | 4 | -353.36 | 723.17 | 1.40 | 0.04 |
| Auto+DTurb+Water+CV+FieldS+Forest+Sunrise+NTurb | 8 | -349.10 | 723.20 | 1.43 | 0.04 |
| Auto+DTurb+Water+FieldS+Forest+Sunrise+NTurb | 7 | -350.22 | 723.28 | 1.51 | 0.04 |
| Auto+DTurb+Water+FieldS+Forest+Sunrise | 6 | -351.30 | 723.31 | 1.54 | 0.04 |
| Auto+DTurb+FieldS+Forest+Sunrise | 5 | -352.43 | 723.43 | 1.65 | 0.04 |
| Auto+Settle+DTurb+FieldS+Forest+Sunrise | 6 | -351.39 | 723.49 | 1.71 | 0.04 |
| Auto+DTurb+Forest+Sunrise+NTurb | 5 | -352.47 | 723.52 | 1.75 | 0.04 |
| Auto+DTurb+Water+CV+Forest+Sunrise+NTurb+Road | 8 | -349.28 | 723.57 | 1.80 | 0.04 |
| Auto+Settle+DTurb+Water+CV+Forest+Sunrise+NTurb | 8 | -349.29 | 723.57 | 1.80 | 0.04 |
| Auto+Settle+FieldS+Forest+Sunrise+NTurb | 6 | -351.44 | 723.58 | 1.81 | 0.04 |
| Auto+Settle+Water+CV+FieldS+Forest+Sunrise | 7 | -350.41 | 723.67 | 1.90 | 0.03 |
| Auto+Settle+Water+FieldS+Forest+Sunrise+NTurb | 7 | -350.43 | 723.71 | 1.94 | 0.03 |
| Auto+Settle+Water+CV+FieldS+Forest+Sunrise+NTurb | 8 | -349.37 | 723.74 | 1.97 | 0.03 |
| ***Autumn migration 2011*** |  |  |  |  |  |
| Auto+CV+Forest+Sunrise+Sunrise^2 | 5 | -1031.70 | 2081.73 | 0.00 | 0.23 |
| Auto+Settle+CV+Forest+Sunrise+Sunrise^2 | 6 | -1030.92 | 2082.26 | 0.52 | 0.18 |
| Auto+CV+Forest+Sunrise | 4 | -1033.12 | 2082.52 | 0.78 | 0.16 |
| Auto+CV+Forest+Sunrise+Sunrise^2+Road | 6 | -1031.12 | 2082.67 | 0.93 | 0.14 |
| Auto+Water+CV+Forest+Sunrise+Sunrise^2 | 6 | -1031.43 | 2083.27 | 1.54 | 0.11 |
| Auto+Settle+CV+Forest+Sunrise | 5 | -1032.54 | 2083.41 | 1.68 | 0.10 |
| Auto+Settle+Water+CV+Forest+Sunrise+Sunrise^2 | 7 | -1030.57 | 2083.64 | 1.91 | 0.09 |
| ***Autumn migration 2012*** |  |  |  |  |  |
| Auto+Sunrise+Sunrise^2 | 3 | -825.68 | 1665.57 | 0.00 | 0.21 |
| Auto+CV+Sunrise+Sunrise^2 | 4 | -824.93 | 1666.12 | 0.56 | 0.16 |
| Auto+Sunrise+Sunrise^2+Road | 4 | -825.18 | 1666.64 | 1.07 | 0.12 |
| Auto+FieldS+Sunrise+Sunrise^2 | 4 | -825.46 | 1667.18 | 1.62 | 0.09 |
| Auto+CV+Sunrise+Sunrise^2+Road | 5 | -824.45 | 1667.24 | 1.67 | 0.09 |
| Auto+Sunrise+Sunrise^2+NTurb | 4 | -825.55 | 1667.38 | 1.81 | 0.08 |
| Auto+Forest+Sunrise+Sunrise^2 | 4 | -825.56 | 1667.40 | 1.83 | 0.08 |
| Auto+DTurb+Sunrise+Sunrise^2 | 4 | -825.64 | 1667.56 | 1.99 | 0.08 |
| Auto+Settle+Sunrise+Sunrise^2 | 4 | -825.64 | 1667.56 | 1.99 | 0.08 |
| ***Winter 2011*** |  |  |  |  |  |
| Auto+Sunrise | 2 | -419.67 | 851.74 | 0.00 | 0.07 |
| Auto | 1 | -420.77 | 851.83 | 0.09 | 0.07 |
| Auto+Water+Sunrise | 3 | -419.02 | 852.57 | 0.83 | 0.05 |
| Auto+Water | 2 | -420.09 | 852.58 | 0.83 | 0.05 |
| Auto+Water+Forest | 3 | -419.20 | 852.94 | 1.19 | 0.04 |
| Auto+Water+Forest+Sunrise | 4 | -418.13 | 852.96 | 1.22 | 0.04 |
| Auto+CV+Sunrise | 3 | -419.26 | 853.05 | 1.31 | 0.04 |
| Auto+CV | 2 | -420.34 | 853.08 | 1.34 | 0.04 |
| Auto+DTurb+Sunrise | 3 | -419.30 | 853.14 | 1.40 | 0.04 |
| Auto+FieldS+Sunrise | 3 | -419.32 | 853.18 | 1.43 | 0.04 |
| Auto+Settle+Sunrise | 3 | -419.32 | 853.18 | 1.44 | 0.04 |
| Auto+Settle | 2 | -420.40 | 853.19 | 1.45 | 0.03 |
| Auto+Sunrise^2 | 2 | -420.40 | 853.20 | 1.45 | 0.03 |
| Auto+FieldS | 2 | -420.40 | 853.20 | 1.45 | 0.03 |
| Auto+DTurb | 2 | -420.41 | 853.23 | 1.49 | 0.03 |
| Auto+Water+CV | 3 | -419.35 | 853.25 | 1.50 | 0.03 |
| Auto+Water+CV+Sunrise | 4 | -418.32 | 853.34 | 1.60 | 0.03 |
| Auto+Water+CV+Forest | 4 | -418.34 | 853.37 | 1.63 | 0.03 |
| Auto+Forest+Sunrise | 3 | -419.43 | 853.40 | 1.66 | 0.03 |
| Auto+Sunrise+NTurb | 3 | -419.43 | 853.40 | 1.66 | 0.03 |
| Auto+Sunrise+Road | 3 | -419.45 | 853.43 | 1.69 | 0.03 |
| Auto+Forest | 2 | -420.54 | 853.48 | 1.74 | 0.03 |
| Auto+NTurb | 2 | -420.54 | 853.49 | 1.75 | 0.03 |
| Auto+Water+CV+Forest+Sunrise | 5 | -417.31 | 853.50 | 1.76 | 0.03 |
| Auto+DTurb+Water | 3 | -419.50 | 853.55 | 1.80 | 0.03 |
| Auto+Road | 2 | -420.57 | 853.55 | 1.80 | 0.03 |
| Auto+DTurb+Water+Sunrise | 4 | -418.43 | 853.55 | 1.81 | 0.03 |
| ***Winter 2012*** |  |  |  |  |  |
| Auto+CV+Sunrise^2 | 3 | -364.47 | 743.48 | 0.00 | 0.12 |
| Auto+CV+Sunrise^2+NTurb | 4 | -363.51 | 743.71 | 0.23 | 0.11 |
| Auto+Sunrise^2 | 2 | -365.74 | 743.87 | 0.40 | 0.10 |
| Auto+Water+Sunrise^2 | 3 | -364.68 | 743.89 | 0.41 | 0.10 |
| Auto+Water+CV+Sunrise^2 | 3 | -363.75 | 744.20 | 0.73 | 0.09 |
| Auto+Water+CV+Sunrise^2+NTurb | 5 | -362.73 | 744.33 | 0.85 | 0.08 |
| Auto+Water+Sunrise^2+NTurb | 4 | -364.14 | 744.98 | 1.50 | 0.06 |
| Auto+CV+Forest+Sunrise^2 | 4 | -364.23 | 745.16 | 1.69 | 0.05 |
| Auto+Sunrise^2+NTurb | 4 | -365.33 | 745.20 | 1.72 | 0.05 |
| Auto+CV+FieldS+Sunrise^2+NTurb | 5 | -363.18 | 745.23 | 1.75 | 0.05 |
| Auto+CV+Sunrise^2+NTurb+Road | 5 | -363.27 | 745.42 | 1.94 | 0.05 |
| Auto+CV+FieldS+Sunrise^2 | 4 | -364.38 | 745.45 | 1.97 | 0.05 |
| Auto+Forest+Sunrise^2 | 3 | -365.46 | 745.47 | 1.99 | 0.05 |
| Auto+CV+Sunrise^2+Road | 4 | -364.39 | 745.48 | 2.00 | 0.05 |

Table S10. Averaged values of function parameters of variables that were present in the best models describing abundance of yellowhammer *Emberiza citrinella*. Explanations of variable abbreviations are given in Table 1. Intercept – a constant which is the expected mean value of dependent variable when all independent variables equal zero. Statistically significant effects are emboldened.

| ***Emberiza citrinella*** | Estimate | SE *adj* | -95 % CI | +95 % CI |
| --- | --- | --- | --- | --- |
| *Spring migration 2011* |  |  |  |  |
| **Intercept** | **-1.345** | **0.401** | **-2.131** | **-0.559** |
| Water | -0.443 | 0.258 | -0.949 | 0.063 |
| **CV** | **0.533** | **0.218** | **0.105** | **0.961** |
| **Forest** | **0.823** | **0.268** | **0.298** | **1.348** |
| Auto | 0.216 | 0.149 | -0.076 | 0.508 |
| NTurb | 0.293 | 0.226 | -0.150 | 0.735 |
| Settle | -0.251 | 0.225 | -0.692 | 0.189 |
| FieldS | 0.097 | 0.208 | -0.311 | 0.505 |
| Road | 0.094 | 0.201 | -0.300 | 0.489 |
| *Spring migration 2012* |  |  |  |  |
| **Intercept** | **-0.794** | **0.247** | **-1.279** | **-0.309** |
| **DTurb** | **0.344** | **0.149** | **0.052** | **0.635** |
| Road | -0.317 | 0.136 | -0.583 | -0.051 |
| **Auto** | **-0.289** | **0.123** | **-0.530** | **-0.048** |
| Forest | 0.152 | 0.138 | -0.119 | 0.422 |
| Sunrise | -0.094 | 0.094 | -0.278 | 0.091 |
| Settle | -0.127 | 0.142 | -0.406 | 0.152 |
| CV | 0.087 | 0.120 | -0.148 | 0.322 |
| NTurb | 0.100 | 0.139 | -0.171 | 0.372 |
| Water | -0.082 | 0.139 | -0.354 | 0.190 |
| *Breeding period 2011* |  |  |  |  |
| **Intercept** | **-1.236** | **0.244** | **-1.714** | **-0.757** |
| **DTurb** | **0.284** | **0.139** | **0.011** | **0.557** |
| CV | 0.223 | 0.133 | -0.037 | 0.483 |
| **Forest** | **0.417** | **0.142** | **0.138** | **0.695** |
| **Auto** | **-0.344** | **0.108** | **-0.556** | **-0.132** |
| NTurb | 0.184 | 0.151 | -0.112 | 0.480 |
| Sunrise^2 | 0.080 | 0.041 | 0.000 | 0.161 |
| Water | -0.139 | 0.157 | -0.448 | 0.170 |
| Sunrise | -0.106 | 0.072 | -0.247 | 0.035 |
| Settle | -0.141 | 0.104 | -0.344 | 0.062 |
| Road | 0.065 | 0.139 | -0.208 | 0.337 |
| *Breeding period 2012* |  |  |  |  |
| **Intercept** | **-0.411** | **0.124** | **-0.654** | **-0.168** |
| DTurb | 0.247 | 0.128 | -0.004 | 0.498 |
| Water | -0.186 | 0.121 | -0.422 | 0.050 |
| CV | 0.152 | 0.102 | -0.048 | 0.353 |
| **Forest** | **0.352** | **0.128** | **0.101** | **0.603** |
| **Sunrise** | **-0.156** | **0.061** | **-0.276** | **-0.037** |
| NTurb | 0.191 | 0.122 | -0.048 | 0.429 |
| Auto | -0.130 | 0.074 | -0.275 | 0.015 |
| Settle | -0.215 | 0.121 | -0.453 | 0.023 |
| FieldS | 0.200 | 0.121 | -0.038 | 0.437 |
| Road | 0.057 | 0.096 | -0.130 | 0.245 |
| *Autumn migration 2011* |  |  |  |  |
| Intercept | -0.417 | 0.270 | -0.947 | 0.113 |
| **CV** | **0.276** | **0.128** | **0.025** | **0.526** |
| **Forest** | **0.433** | **0.129** | **0.180** | **0.687** |
| **Sunrise** | **-0.161** | **0.040** | **-0.239** | **-0.083** |
| Sunrise^2 | -0.141 | 0.081 | -0.300 | 0.018 |
| **Auto** | **0.306** | **0.045** | **0.218** | **0.395** |
| Settle | 0.152 | 0.123 | -0.090 | 0.394 |
| Road | -0.140 | 0.129 | -0.393 | 0.114 |
| Water | -0.112 | 0.144 | -0.394 | 0.170 |
| *Autumn migration 2012* |  |  |  |  |
| Intercept | -0.100 | 0.264 | -0.616 | 0.417 |
| **Sunrise** | **-0.167** | **0.051** | **-0.266** | **-0.067** |
| **Sunrise^2** | **-0.408** | **0.090** | **-0.584** | **-0.231** |
| **Auto** | **0.164** | **0.060** | **0.047** | **0.281** |
| CV | 0.147 | 0.120 | -0.088 | 0.382 |
| Road | -0.119 | 0.120 | -0.353 | 0.116 |
| FieldS | 0.085 | 0.128 | -0.165 | 0.335 |
| NTurb | -0.061 | 0.122 | -0.301 | 0.179 |
| Forest | 0.057 | 0.119 | -0.177 | 0.291 |
| DTurb | 0.032 | 0.120 | -0.204 | 0.268 |
| Settle | 0.032 | 0.122 | -0.208 | 0.271 |
| *Winter 2011* |  |  |  |  |
| **Intercept** | **-1.315** | **0.481** | **-2.258** | **-0.371** |
| Sunrise | 0.116 | 0.068 | -0.016 | 0.249 |
| **Auto** | **5.362** | **0.429** | **4.521** | **6.203** |
| Water | -0.519 | 0.287 | -1.081 | 0.043 |
| Forest | 0.405 | 0.216 | -0.019 | 0.829 |
| CV | -0.369 | 0.270 | -0.898 | 0.161 |
| DTurb | 0.345 | 0.251 | -0.147 | 0.838 |
| FieldS | -0.284 | 0.324 | -0.918 | 0.351 |
| Settle | -0.282 | 0.330 | -0.929 | 0.365 |
| Sunrise^2 | 0.124 | 0.138 | -0.147 | 0.396 |
| NTurb | -0.233 | 0.339 | -0.898 | 0.432 |
| Road | -0.213 | 0.324 | -0.848 | 0.423 |
| *Winter 2012* |  |  |  |  |
| Intercept | -0.484 | 0.477 | -1.419 | 0.451 |
| CV | 0.471 | 0.278 | -0.073 | 1.015 |
| **Sunrise^2** | **-0.418** | **0.100** | **-0.614** | **-0.222** |
| **Auto** | **0.347** | **0.146** | **0.060** | **0.634** |
| NTurb | 0.364 | 0.279 | -0.183 | 0.911 |
| Water | -0.381 | 0.280 | -0.930 | 0.168 |
| Forest | -0.196 | 0.272 | -0.729 | 0.338 |
| FieldS | -0.181 | 0.289 | -0.747 | 0.386 |
| Road | -0.144 | 0.264 | -0.660 | 0.373 |

Table S11. Best models describing abundance of yellow wagtail *Motacilla flava* in different seasons and years. For each model the number of explanatory variables (k), log-likelihood, corrected Akaike (AICc), difference between Akaike of a given model and best model (Δ), and model weight (*w*). Explanations of variable abbreviations are given in Table 1. Variable describing the effect of spatial autocorrelation (Auto) were included into each model. Explanations of variable abbreviations are given in Table 1.

| **Model** | **k** | **logLik** | **AICc** | **delta** | **weight** |
| --- | --- | --- | --- | --- | --- |
| ***Motacilla flava*** |  |  |  |  |  |
| ***Spring migration 2011*** |  |  |  |  |  |
| Auto+Water+Sunrise+NTurb | 4 | -84.49 | 186.43 | 0.00 | 0.10 |
| Auto+Water+NTurb | 3 | -85.85 | 186.83 | 0.40 | 0.08 |
| Auto+Water+Sunrise+Sunrise^2+NTurb | 5 | -83.62 | 187.08 | 0.66 | 0.07 |
| Auto+Settle+Water+Sunrise+NTurb | 5 | -83.76 | 187.36 | 0.94 | 0.06 |
| Auto+Sunrise+NTurb | 3 | -86.19 | 187.50 | 1.08 | 0.06 |
| Auto+Water+Sunrise | 3 | -86.23 | 187.59 | 1.16 | 0.05 |
| Auto+Settle+Water+Sunrise+Sunrise^2+NTurb | 6 | -82.67 | 187.60 | 1.18 | 0.05 |
| Auto+Water+Sunrise+NTurb+Road | 5 | -83.96 | 187.76 | 1.33 | 0.05 |
| Auto+Settle+Water+NTurb | 4 | -85.16 | 187.78 | 1.35 | 0.05 |
| Auto+Water+Sunrise+Sunrise^2+NTurb+Road | 6 | -82.81 | 187.89 | 1.46 | 0.05 |
| Auto+Sunrise | 2 | -87.54 | 187.92 | 1.49 | 0.05 |
| Auto+Water+Sunrise+Sunrise^2 | 4 | -85.29 | 188.03 | 1.60 | 0.04 |
| Auto+Water+NTurb+Road | 4 | -85.29 | 188.04 | 1.61 | 0.04 |
| Auto+Forest+Sunrise | 3 | -86.49 | 188.10 | 1.67 | 0.04 |
| Auto+Sunrise+Sunrise^2+NTurb | 4 | -85.34 | 188.14 | 1.71 | 0.04 |
| Auto+NTurb | 2 | -87.66 | 188.16 | 1.73 | 0.04 |
| Auto+Water+FieldS+Sunrise+NTurb | 2 | -84.18 | 188.20 | 1.77 | 0.04 |
| Auto+Water | 5 | -87.73 | 188.29 | 1.86 | 0.04 |
| Auto+Sunrise+Sunrise^2 | 3 | -86.63 | 188.38 | 1.95 | 0.04 |
| ***Spring migration 2012*** |  |  |  |  |  |
| Auto | 1 | -46.57 | 104.04 | 0.00 | 0.16 |
| Auto+Forest | 2 | -45.57 | 104.44 | 0.40 | 0.13 |
| Auto+Sunrise^2 | 2 | -45.76 | 104.81 | 0.77 | 0.11 |
| Auto+Forest+Road | 3 | -44.68 | 105.12 | 1.07 | 0.09 |
| Auto+Forest+Sunrise^2+Road | 4 | -43.48 | 105.25 | 1.21 | 0.09 |
| Auto+Water+Forest | 3 | -44.80 | 105.36 | 1.31 | 0.08 |
| Auto+Sunrise^2+Road | 3 | -44.81 | 105.37 | 1.33 | 0.08 |
| Auto+Road | 2 | -46.12 | 105.52 | 1.48 | 0.07 |
| Auto+Forest+Sunrise^2 | 3 | -44.98 | 105.70 | 1.66 | 0.07 |
| Auto+Forest+NTurb | 3 | -45.00 | 105.75 | 1.71 | 0.07 |
| Auto+Water+FieldS+Forest+Sunrise^2+NTurb | 6 | -41.21 | 106.02 | 1.98 | 0.06 |
| ***Breeding period 2011*** |  |  |  |  |  |
| Auto+Settle+DTurb+FieldS+Forest+Sunrise+Sunrise^2 | 7 | -424.57 | 871.98 | 0.00 | 0.25 |
| Auto+Settle+DTurb+Water+FieldS+Forest+Sunrise+Sunrise^2 | 8 | -423.93 | 872.87 | 0.89 | 0.16 |
| Auto+Settle+DTurb+FieldS+Forest+Sunrise^2 | 6 | -426.30 | 873.30 | 1.32 | 0.13 |
| Auto+DTurb+FieldS+Forest+Sunrise+Sunrise^2 | 6 | -426.30 | 873.31 | 1.33 | 0.13 |
| Auto+Settle+DTurb+FieldS+Forest+Sunrise+Sunrise^2+NTurb | 8 | -424.24 | 873.49 | 1.51 | 0.12 |
| Auto+Settle+DTurb+FieldS+Forest+Sunrise+Sunrise^2+Road | 8 | -424.34 | 873.67 | 1.69 | 0.11 |
| Auto+Settle+DTurb+Water+FieldS+Forest+Sunrise+Sunrise^2+NTurb | 9 | -423.33 | 873.83 | 1.85 | 0.10 |
| ***Breeding period 2012*** |  |  |  |  |  |
| Auto+Settle+CV+Forest | 4 | -408.63 | 833.72 | 0.00 | 0.14 |
| Auto+Settle+CV+Forest+Sunrise^2 | 5 | -407.71 | 833.99 | 0.27 | 0.12 |
| Auto+Settle+CV+Forest+Sunrise^2+Road | 6 | -406.86 | 834.43 | 0.71 | 0.10 |
| Auto+Settle+CV+Forest+Road | 5 | -407.96 | 834.49 | 0.77 | 0.09 |
| Auto+Settle+CV+Forest+NTurb | 5 | -408.12 | 834.81 | 1.09 | 0.08 |
| Auto+Settle+CV+Forest+Sunrise | 5 | -408.28 | 835.14 | 1.42 | 0.07 |
| Auto+Settle+Forest+NTurb | 4 | -409.35 | 835.16 | 1.44 | 0.07 |
| Auto+Settle+CV+FieldS+Forest | 5 | -408.34 | 835.26 | 1.54 | 0.06 |
| Auto+Settle+CV+Forest+Sunrise^2+NTurb | 6 | -407.34 | 835.38 | 1.66 | 0.06 |
| Auto+Settle+Forest | 3 | -410.53 | 835.42 | 1.70 | 0.06 |
| Auto+Settle+Water+CV+Forest | 5 | -408.42 | 835.42 | 1.71 | 0.06 |
| Auto+Settle+Water+CV+Forest+Sunrise^2 | 6 | -407.39 | 835.48 | 1.76 | 0.06 |
| Auto+Settle+CV+Forest+Sunrise+Sunrise^2 | 6 | -407.51 | 835.71 | 2.00 | 0.05 |
| ***Autumn migration 2011*** |  |  |  |  |  |
| Auto+Water+FieldS+Sunrise | 4 | -257.61 | 531.91 | 0.00 | 0.20 |
| Auto+Water+Sunrise | 3 | -258.76 | 532.05 | 0.14 | 0.18 |
| Auto+Water+Sunrise+NTurb | 4 | -258.09 | 532.88 | 0.97 | 0.12 |
| Auto+Water+CV+FieldS+Sunrise | 5 | -257.05 | 532.96 | 1.06 | 0.12 |
| Auto+Water+Sunrise+Sunrise^2 | 4 | -258.26 | 533.21 | 1.31 | 0.10 |
| Auto+Water+FieldS+Sunrise+NTurb | 5 | -257.18 | 533.24 | 1.33 | 0.10 |
| Auto+Water+CV+Sunrise | 4 | -258.33 | 533.35 | 1.45 | 0.10 |
| Auto+Water+FieldS+Sunrise+Sunrise^2 | 5 | -257.48 | 533.83 | 1.93 | 0.08 |
| ***Autumn migration 2012*** |  |  |  |  |  |
| Auto+DTurb+Water+Sunrise+Sunrise^2+NTurb+Road | 7 | -229.93 | 482.82 | 0.00 | 0.42 |
| Auto+Settle+DTurb+Water+Sunrise+Sunrise^2+NTurb+Road | 8 | -229.35 | 483.84 | 1.02 | 0.25 |
| Auto+DTurb+Water+Sunrise+Sunrise^2+Road | 6 | -231.89 | 484.58 | 1.76 | 0.17 |
| Auto+DTurb+Water+CV+Sunrise+Sunrise^2+NTurb+Road | 8 | -229.79 | 484.70 | 1.89 | 0.16 |

Table S12. Averaged values of function parameters of variables that were present in the best models describing abundance of yellow wagtail *Motacilla flava*. Explanations of variable abbreviations are given in Table 1. Intercept – a constant which is the expected mean value of dependent variable when all independent variables equal zero. Statistically significant effects are emboldened.

| ***Motacilla flava*** | Estimate | SE *adj* | -95 % CI | +95 % CI |
| --- | --- | --- | --- | --- |
| *Spring migration 2011* |  |  |  |  |
| **Intercept** | **-1.460** | **0.336** | **-2.119** | **-0.800** |
| **Water** | **-0.387** | **0.104** | **-0.590** | **-0.184** |
| **Sunrise** | **-0.353** | **0.133** | **-0.613** | **-0.093** |
| **NTurb** | **0.368** | **0.107** | **0.158** | **0.578** |
| **Auto** | **-0.448** | **0.171** | **-0.783** | **-0.113** |
| **Sunrise^2** | **-0.361** | **0.183** | **-0.719** | **-0.003** |
| **Settle** | **0.258** | **0.008** | **0.241** | **0.274** |
| Road | -0.244 | 0.175 | -0.588 | 0.099 |
| **Forest** | **-0.313** | **0.002** | **-0.318** | **-0.309** |
| **FieldS** | **-0.163** | **0.002** | **-0.167** | **-0.158** |
| *Spring migration 2012* |  |  |  |  |
| Intercept | -2.002 | 1.036 | -4.033 | 0.029 |
| Auto | -1.683 | 2.769 | -7.109 | 3.744 |
| Forest | -0.433 | 0.282 | -0.986 | 0.120 |
| Sunrise^2 | -0.447 | 0.282 | -1.000 | 0.106 |
| Road | -0.339 | 0.228 | -0.787 | 0.108 |
| Water | 0.352 | 0.241 | -0.119 | 0.823 |
| NTurb | -0.443 | 0.345 | -1.119 | 0.233 |
| FieldS | 0.431 | 0.222 | -0.005 | 0.866 |
| *Breeding period 2011* |  |  |  |  |
| Intercept | -0.100 | 0.100 | -0.297 | 0.096 |
| Settle | 0.182 | 0.095 | -0.005 | 0.369 |
| **DTurb** | **-0.225** | **0.094** | **-0.411** | **-0.040** |
| **FieldS** | **0.266** | **0.090** | **0.088** | **0.443** |
| **Forest** | **-0.445** | **0.105** | **-0.650** | **-0.240** |
| Sunrise | -0.132 | 0.069 | -0.267 | 0.004 |
| **Sunrise^2** | **-0.119** | **0.059** | **-0.235** | **-0.004** |
| **Auto** | **-0.483** | **0.238** | **-0.949** | **-0.016** |
| Water | -0.101 | 0.085 | -0.267 | 0.064 |
| NTurb | 0.079 | 0.086 | -0.089 | 0.248 |
| Road | 0.060 | 0.086 | -0.109 | 0.228 |
| *Breeding period 2012* |  |  |  |  |
| Intercept | -0.342 | 0.203 | -0.741 | 0.056 |
| **Settle** | **0.308** | **0.121** | **0.071** | **0.545** |
| **CV** | **-0.242** | **0.119** | **-0.475** | **-0.009** |
| **Forest** | **-0.279** | **0.127** | **-0.528** | **-0.030** |
| Auto | -0.009 | 0.379 | -0.753 | 0.734 |
| Sunrise^2 | 0.136 | 0.099 | -0.057 | 0.329 |
| Road | 0.157 | 0.124 | -0.087 | 0.401 |
| NTurb | 0.144 | 0.131 | -0.112 | 0.400 |
| Sunrise | -0.046 | 0.060 | -0.164 | 0.072 |
| FieldS | 0.093 | 0.122 | -0.146 | 0.332 |
| Water | -0.096 | 0.134 | -0.359 | 0.166 |
| *Autumn migration 2011* |  |  |  |  |
| **Intercept** | **-0.718** | **0.306** | **-1.317** | **-0.119** |
| **Water** | **-0.600** | **0.125** | **-0.845** | **-0.355** |
| FieldS | 0.179 | 0.120 | -0.057 | 0.415 |
| **Sunrise** | **-0.593** | **0.083** | **-0.756** | **-0.429** |
| Auto | 0.381 | 0.420 | -0.442 | 1.203 |
| NTurb | 0.127 | 0.120 | -0.108 | 0.362 |
| CV | -0.109 | 0.108 | -0.320 | 0.102 |
| Sunrise^2 | 0.071 | 0.091 | -0.107 | 0.249 |
| *Autumn migration 2012* |  |  |  |  |
| **Intercept** | **-1.309** | **0.592** | **-2.469** | **-0.149** |
| **DTurb** | **-0.284** | **0.121** | **-0.521** | **-0.047** |
| **Water** | **-0.297** | **0.129** | **-0.549** | **-0.046** |
| **Sunrise** | **-0.277** | **0.104** | **-0.481** | **-0.073** |
| **Sunrise^2** | **-0.374** | **0.120** | **-0.608** | **-0.140** |
| **NTurb** | **0.248** | **0.126** | **0.001** | **0.494** |
| **Road** | **-0.371** | **0.136** | **-0.638** | **-0.104** |
| **Auto** | **0.718** | **0.316** | **0.099** | **1.338** |
| Settle | 0.140 | 0.129 | -0.112 | 0.393 |
| CV | -0.065 | 0.118 | -0.296 | 0.166 |

Table S13. Best models describing abundance of lapwing *Vanellus vanellus* in different seasons and years. For each model the number of explanatory variables (k), log-likelihood, corrected Akaike (AICc), difference between Akaike of a given model and best model (Δ), and model weight (*w*). Explanations of variable abbreviations are given in Table 1. Variable describing the effect of spatial autocorrelation (Auto) were included into each model. Explanations of variable abbreviations are given in Table 1.

| **Model** | **k** | **logLik** | **AICc** | **delta** | **weight** |
| --- | --- | --- | --- | --- | --- |
| ***Vanellus vanellus*** |  |  |  |  |  |
| ***Spring migration 2011*** |  |  |  |  |  |
| Auto+Water+CV+FieldS+NTurb | 5 | -25.66 | 70.67 | 0.00 | 0.28 |
| Auto+Settle+Water+CV+FieldS+NTurb | 6 | -24.83 | 71.32 | 0.65 | 0.20 |
| Auto+DTurb+Water+CV+FieldS+NTurb | 6 | -25.17 | 72.00 | 1.33 | 0.14 |
| Auto+Water+CV+FieldS+Sunrise+NTurb | 6 | -25.21 | 72.08 | 1.41 | 0.14 |
| Auto+Water+CV+FieldS+Sunrise^2+NTurb | 6 | -25.32 | 72.30 | 1.62 | 0.12 |
| Auto+Settle+CV+FieldS+Forest+NTurb | 6 | -25.41 | 72.48 | 1.81 | 0.11 |
| ***Spring migration 2012*** |  |  |  |  |  |
| Auto+Settle+DTurb+Water+FieldS+Forest+Sunrise+Road | 8 | -15.05 | 56.48 | 0.00 | 0.19 |
| Auto+DTurb+FieldS+Forest+Sunrise+NTurb+Road | 7 | -16.49 | 56.98 | 0.49 | 0.15 |
| Auto+FieldS+Forest+Sunrise+Sunrise^2+NTurb+Road | 7 | -16.69 | 57.38 | 0.90 | 0.12 |
| Auto+Settle+DTurb+FieldS+Forest+Sunrise+NTurb+Road | 8 | -15.51 | 57.41 | 0.93 | 0.12 |
| Auto+Settle+DTurb+Water+FieldS+Forest+Sunrise+Sunrise^2+Road | 9 | -14.38 | 57.56 | 1.07 | 0.11 |
| Auto+DTurb+FieldS+Forest+Sunrise+Sunrise^2+NTurb+Road | 8 | -15.62 | 57.63 | 1.15 | 0.11 |
| Auto+DTurb+Water+FieldS+Forest+Sunrise+NTurb+Road | 8 | -15.75 | 57.89 | 1.41 | 0.10 |
| Auto+FieldS+Forest+Sunrise+NTurb+Road | 6 | -18.13 | 57.92 | 1.43 | 0.09 |
| ***Breeding period 2011*** |  |  |  |  |  |
| Auto+FieldS+Forest+Nturb | 4 | -52.46 | 121.61 | 0.00 | 0.17 |
| Auto+FieldS+Forest+Nturb+Road | 5 | -51.54 | 121.95 | 0.35 | 0.14 |
| Auto+CV+FieldS+Forest+Sunrise^2+Nturb+Road | 7 | -49.76 | 122.81 | 1.20 | 0.09 |
| Auto+FieldS+Forest+Sunrise^2+Nturb+Road | 6 | -50.93 | 122.92 | 1.32 | 0.09 |
| Auto+Water+FieldS+Forest+Nturb | 5 | -52.03 | 122.94 | 1.33 | 0.09 |
| Auto+CV+FieldS+Forest+Nturb | 5 | -52.14 | 123.15 | 1.54 | 0.08 |
| Auto+FieldS+Forest+Sunrise^2+Nturb | 5 | -52.24 | 123.36 | 1.75 | 0.07 |
| Auto+Settle+FieldS+Forest+Nturb | 5 | -52.25 | 123.38 | 1.77 | 0.07 |
| Auto+FieldS+Forest+Sunrise+Nturb+Road | 6 | -51.16 | 123.39 | 1.78 | 0.07 |
| Auto+FieldS+Forest+Sunrise+Nturb | 5 | -52.29 | 123.45 | 1.84 | 0.07 |
| Auto+CV+FieldS+Forest+Nturb+Road | 6 | -51.26 | 123.59 | 1.99 | 0.06 |
| ***Autumn migration 2011*** |  |  |  |  |  |
| Auto+DTurb+Sunrise+Sunrise^2 | 4 | -131.26 | 279.36 | 0.00 | 0.35 |
| Auto+Water+Sunrise+Sunrise^2+Road | 5 | -131.18 | 281.42 | 2.06 | 0.13 |
| Auto+Sunrise+Sunrise^2+NTurb+Road | 5 | -131.30 | 281.67 | 2.31 | 0.11 |
| Auto+CV+Sunrise+Sunrise^2 | 4 | -132.60 | 282.05 | 2.70 | 0.09 |
| Auto+DTurb+Water+CV+Sunrise+Sunrise^2+NTurb | 7 | -129.27 | 282.11 | 2.76 | 0.09 |
| Auto+Water+Sunrise+Sunrise^2+NTurb | 5 | -131.71 | 282.48 | 3.13 | 0.07 |
| Auto+Water+CV+Sunrise+Sunrise^2+NTurb | 6 | -130.92 | 283.13 | 3.78 | 0.05 |
| Auto+Water+CV+Forest+Sunrise+Sunrise^2 | 6 | -131.02 | 283.33 | 3.98 | 0.05 |
| Auto+DTurb+Water+Sunrise+Sunrise^2+NTurb+Road | 7 | -129.88 | 283.34 | 3.98 | 0.05 |

Table S14. Averaged values of function parameters of variables that were present in the best models describing abundance of lapwing *Vanellus vanellus*. Explanations of variable abbreviations are given in Table 1. Intercept – a constant which is the expected mean value of dependent variable when all independent variables equal zero. Statistically significant effects are emboldened.

| ***Vanellus vanellus*** | Estimate | SE *adj* | -95 % CI | +95 % CI |
| --- | --- | --- | --- | --- |
| *Spring migration 2011* |  |  |  |  |
| **Intercept** | **-6.806** | **1.878** | **-10.487** | **-3.126** |
| **Water** | **-1.508** | **0.555** | **-2.596** | **-0.420** |
| **CV** | **-3.717** | **1.255** | **-6.178** | **-1.257** |
| **FieldS** | **1.971** | **0.718** | **0.565** | **3.378** |
| **NTurb** | **-2.527** | **1.036** | **-4.557** | **-0.497** |
| Auto | 0.772 | 0.804 | -0.805 | 2.348 |
| Settle | -1.387 | 0.936 | -3.222 | 0.448 |
| DTurb | 1.006 | 1.117 | -1.183 | 3.195 |
| Sunrise | -0.320 | 0.264 | -0.837 | 0.197 |
| Sunrise^2 | -0.307 | 0.368 | -1.027 | 0.413 |
| Forest | -1.065 | 0.549 | -2.142 | 0.012 |
| *Spring migration 2012* |  |  | 0.000 | 0.000 |
| Intercept | -383.000 | 729200.000 | -1429615.000 | 1428849.000 |
| Settle | 23.880 | 44120.000 | -86451.320 | 86499.080 |
| DTurb | 10.850 | 21600.000 | -42325.150 | 42346.850 |
| Water | -16.720 | 30640.000 | -60071.120 | 60037.680 |
| FieldS | 198.000 | 378500.000 | -741662.000 | 742058.000 |
| Forest | -285.500 | 546200.000 | -1070837.500 | 1070266.500 |
| **Sunrise** | **-0.949** | **0.476** | **-1.883** | **-0.015** |
| Road | 70.310 | 132100.000 | -258845.690 | 258986.310 |
| Auto | -4.059 | 2.088 | -8.151 | 0.033 |
| NTurb | -0.868 | 0.749 | -2.336 | 0.600 |
| Sunrise^2 | -0.220 | 0.416 | -1.035 | 0.595 |
| *Breeding period 2011* |  |  |  |  |
| **Intercept** | **-5.401** | **1.225** | **-7.803** | **-3.000** |
| FieldS | 1.080 | 0.579 | -0.054 | 2.214 |
| **Forest** | **-1.615** | **0.740** | **-3.064** | **-0.166** |
| **NTurb** | **-3.114** | **0.974** | **-5.023** | **-1.204** |
| Auto | -0.496 | 1.008 | -2.471 | 1.479 |
| Road | 1.102 | 0.714 | -0.298 | 2.501 |
| CV | -0.458 | 0.448 | -1.337 | 0.420 |
| Sunrise^2 | 0.669 | 0.589 | -0.485 | 1.822 |
| Water | -0.436 | 0.473 | -1.364 | 0.492 |
| Settle | 0.334 | 0.481 | -0.608 | 1.275 |
| Sunrise | -0.231 | 0.321 | -0.860 | 0.398 |
| *Autumn migration 2011* |  |  |  |  |
| **Intercept** | **-185.505** | **23.546** | **-231.655** | **-139.354** |
| DTurb | -1.038 | 18.926 | -38.133 | 36.057 |
| **Sunrise** | **0.778** | **0.219** | **0.348** | **1.207** |
| **Sunrise^2** | **36.350** | **3.830** | **28.843** | **43.856** |
| Auto | 4.123 | 2.320 | -0.424 | 8.670 |
| Water | -44.436 | 24.114 | -91.700 | 2.828 |
| **Road** | **12.893** | **4.371** | **4.327** | **21.460** |
| NTurb | 11.958 | 17.109 | -21.575 | 45.490 |
| CV | -8.253 | 19.024 | -45.540 | 29.035 |
| **Forest** | **-30.247** | **7.892** | **-45.715** | **-14.779** |

Table S15. Best models describing abundance of common whitethroat *Sylvia communis* in different seasons and years. For each model the number of explanatory variables (k), log-likelihood, corrected Akaike (AICc), difference between Akaike of a given model and best model (Δ), and model weight (*w*). Explanations of variable abbreviations are given in Table 1. Variable describing the effect of spatial autocorrelation (Auto) were included into each model. Explanations of variable abbreviations are given in Table 1.

| **Model** | **k** | **logLik** | **AICc** | **delta** | **weight** |
| --- | --- | --- | --- | --- | --- |
| ***Sylvia communis*** |  |  |  |  |  |
| ***Spring migration 2011*** |  |  |  |  |  |
| Auto+Settle | 2 | -18.31 | 48.62 | 0.00 | 0.35 |
| Auto | 1 | -19.87 | 49.03 | 0.41 | 0.28 |
| Auto+Road | 2 | -18.92 | 49.84 | 1.22 | 0.19 |
| Auto+Settle+Forest | 3 | -17.48 | 49.86 | 1.24 | 0.19 |
| ***Spring migration 2012*** |  |  |  |  |  |
| Auto | 1 | -10.50 | 30.29 | 0.00 | 0.32 |
| Auto+NTurb | 2 | -9.57 | 31.14 | 0.85 | 0.21 |
| Auto+Settle | 2 | -9.62 | 31.24 | 0.95 | 0.20 |
| Auto+FieldS | 2 | -9.92 | 31.84 | 1.54 | 0.15 |
| Auto+Settle+NTurb | 3 | -8.65 | 32.19 | 1.89 | 0.12 |
| ***Breeding period 2011*** |  |  |  |  |  |
| Auto+DTurb+Forest+Sunrise | 4 | -262.58 | 541.61 | 0.00 | 0.08 |
| Auto+DTurb+Forest | 3 | -263.66 | 541.68 | 0.07 | 0.08 |
| Auto+DTurb+Sunrise | 3 | -263.77 | 541.90 | 0.29 | 0.07 |
| Auto+DTurb | 2 | -264.97 | 542.20 | 0.59 | 0.06 |
| Auto+DTurb+FieldS+Forest+Sunrise | 5 | -261.89 | 542.34 | 0.73 | 0.06 |
| Auto+DTurb+CV+Forest+Sunrise | 5 | -262.00 | 542.58 | 0.97 | 0.05 |
| Auto+Settle+FieldS+Sunrise | 4 | -263.09 | 542.65 | 1.03 | 0.05 |
| Auto+DTurb+FieldS+Sunrise | 4 | -263.12 | 542.70 | 1.09 | 0.05 |
| Auto+DTurb+CV+Forest | 4 | -263.23 | 542.91 | 1.30 | 0.04 |
| Auto+DTurb+FieldS+Forest | 4 | -263.23 | 542.92 | 1.31 | 0.04 |
| Auto+DTurb+Forest+Sunrise^2 | 4 | -263.35 | 543.15 | 1.54 | 0.04 |
| Auto+DTurb+CV+Sunrise | 4 | -263.37 | 543.20 | 1.58 | 0.04 |
| Auto+DTurb+Sunrise+NTurb | 4 | -263.40 | 543.26 | 1.65 | 0.04 |
| Auto+Settle+DTurb+FieldS+Sunrise | 5 | -262.35 | 543.26 | 1.65 | 0.04 |
| Auto+DTurb+CV+FieldS+Forest+Sunrise | 6 | -261.35 | 543.41 | 1.79 | 0.03 |
| Auto+DTurb+Forest+Sunrise+Road | 5 | -262.45 | 543.47 | 1.85 | 0.03 |
| Auto+DTurb+Sunrise+Road | 4 | -263.51 | 543.47 | 1.86 | 0.03 |
| Auto+DTurb+Forest+Sunrise+NTurb | 5 | -262.46 | 543.49 | 1.88 | 0.03 |
| Auto+DTurb+Water+Forest+Sunrise | 5 | -262.47 | 543.51 | 1.90 | 0.03 |
| Auto+DTurb+FieldS | 3 | -264.58 | 543.51 | 1.90 | 0.03 |
| Auto+Settle+DTurb+FieldS+Forest+Sunrise | 6 | -261.42 | 543.54 | 1.93 | 0.03 |
| Auto+DTurb+Forest+Sunrise+Sunrise^2 | 5 | -262.50 | 543.57 | 1.95 | 0.03 |
| Auto+DTurb+Sunrise^2 | 3 | -264.63 | 543.61 | 2.00 | 0.03 |
| ***Breeding period 2012*** |  |  |  |  |  |
| Auto+DTurb+Sunrise^2+Road | 4 | -265.78 | 548.02 | 0.00 | 0.10 |
| Auto+Settle+Sunrise^2+Road | 4 | -265.83 | 548.11 | 0.09 | 0.10 |
| Auto+Settle+DTurb+Sunrise^2+Road | 5 | -265.12 | 548.82 | 0.79 | 0.07 |
| Auto+DTurb+Water+Sunrise^2+Road | 5 | -265.15 | 548.88 | 0.86 | 0.07 |
| Auto+DTurb+Forest+Sunrise^2+Road | 5 | -265.19 | 548.95 | 0.93 | 0.06 |
| Auto+Settle+Sunrise+Sunrise^2+Road | 5 | -265.21 | 548.99 | 0.97 | 0.06 |
| Auto+DTurb+Sunrise+Sunrise^2+Road | 5 | -265.26 | 549.10 | 1.07 | 0.06 |
| Auto+DTurb+Road | 3 | -267.47 | 549.30 | 1.27 | 0.05 |
| Auto+Settle+Sunrise^2 | 3 | -267.54 | 549.44 | 1.42 | 0.05 |
| Auto+Settle+Water+Sunrise^2+Road | 5 | -265.57 | 549.72 | 1.69 | 0.04 |
| Auto+Settle+Sunrise^2+NTurb+Road | 5 | -265.60 | 549.76 | 1.74 | 0.04 |
| Auto+DTurb+FieldS+Sunrise^2+Road | 5 | -265.61 | 549.79 | 1.77 | 0.04 |
| Auto+Settle+DTurb+Sunrise+Sunrise^2+Road | 6 | -264.56 | 549.83 | 1.81 | 0.04 |
| Auto+Settle+DTurb+Water+Sunrise^2+Road | 6 | -264.60 | 549.90 | 1.88 | 0.04 |
| Auto+DTurb+Sunrise^2+NTurb+Road | 5 | -265.68 | 549.94 | 1.92 | 0.04 |
| Auto+Sunrise^2+Road | 3 | -267.81 | 549.97 | 1.95 | 0.04 |
| Auto+DTurb+Water+Sunrise+Sunrise^2+Road | 6 | -264.64 | 549.98 | 1.95 | 0.04 |
| Auto+DTurb+Forest+Sunrise+Sunrise^2+Road | 6 | -264.66 | 550.01 | 1.99 | 0.04 |
| ***Autumn migration 2011*** |  |  |  |  |  |
| Auto+DTurb | 2 | -57.55 | 125.54 | 0 | 0.23 |
| Auto+DTurb+Sunrise | 3 | -56.85 | 126.32 | 0.77 | 0.16 |
| Auto | 1 | -59.08 | 126.45 | 0.91 | 0.15 |
| Auto+Sunrise | 2 | -58.37 | 127.18 | 1.64 | 0.1 |
| Auto+DTurb+Water | 3 | -57.37 | 127.35 | 1.81 | 0.09 |
| Auto+Settle+DTurb | 3 | -57.43 | 127.47 | 1.92 | 0.09 |
| Auto+DTurb+Forest | 3 | -57.43 | 127.47 | 1.93 | 0.09 |
| Auto+DTurb+NTurb | 3 | -57.45 | 127.52 | 1.98 | 0.09 |
| ***Autumn migration 2012*** |  |  |  |  |  |
| Auto+Settle+NTurb+Road | 4 | -19.26 | 55.97 | 0.00 | 0.22 |
| Auto+Settle+Sunrise+NTurb+Road | 5 | -18.70 | 57.24 | 1.26 | 0.12 |
| Auto+Settle+CV+NTurb+Road | 5 | -18.76 | 57.35 | 1.37 | 0.11 |
| Auto+Settle+Forest+NTurb+Road | 5 | -18.77 | 57.37 | 1.40 | 0.11 |
| Auto+Settle+Water+NTurb+Road | 5 | -18.93 | 57.70 | 1.72 | 0.09 |
| Auto+DTurb+Forest+NTurb+Road | 5 | -18.96 | 57.76 | 1.78 | 0.09 |
| Auto+NTurb+Road | 3 | -21.36 | 57.84 | 1.86 | 0.09 |
| Auto+DTurb+Forest+Road | 4 | -20.21 | 57.88 | 1.91 | 0.09 |
| Auto+Settle+Forest+Road | 4 | -20.26 | 57.97 | 1.99 | 0.08 |

Table S16. Averaged values of function parameters of variables that were present in the best models describing abundance of common whitethroat *Sylvia communis*. Explanations of variable abbreviations are given in Table 1. Intercept – a constant which is the expected mean value of dependent variable when all independent variables equal zero. Statistically significant effects are emboldened.

| ***Sylvia communis*** | Estimate | SE *adj* | -95 % CI | +95 % CI |
| --- | --- | --- | --- | --- |
| *Spring migration 2011* |  |  |  |  |
| **Intercept** | **-2.453** | **0.817** | **-4.054** | **-0.852** |
| Settle | -0.985 | 0.649 | -2.256 | 0.286 |
| Auto | 2.545 | 2.116 | -1.602 | 6.693 |
| Road | 0.672 | 0.527 | -0.362 | 1.705 |
| Forest | -0.647 | 0.547 | -1.718 | 0.425 |
| *Spring migration 2012* |  |  |  |  |
| Intercept | -3.452 | 2.594 | -8.536 | 1.633 |
| Auto | -0.021 | 2.523 | -4.966 | 4.925 |
| NTurb | -0.980 | 0.870 | -2.686 | 0.726 |
| Settle | -0.869 | 0.778 | -2.393 | 0.655 |
| FieldSize | -0.780 | 0.842 | -2.431 | 0.870 |
| *Breeding period 2011* |  |  |  |  |
| **Intercept** | **-1.090** | **0.174** | **-1.431** | **-0.750** |
| **DTurb** | **0.383** | **0.167** | **0.056** | **0.710** |
| Forest | -0.246 | 0.158 | -0.555 | 0.063 |
| Sunrise | -0.161 | 0.103 | -0.363 | 0.042 |
| Auto | 0.560 | 0.447 | -0.315 | 1.435 |
| FieldSize | 0.196 | 0.161 | -0.120 | 0.512 |
| CV | -0.131 | 0.132 | -0.389 | 0.127 |
| Settle | -0.245 | 0.179 | -0.596 | 0.106 |
| Sunrise^2 | -0.051 | 0.079 | -0.206 | 0.104 |
| NTurb | 0.105 | 0.155 | -0.200 | 0.410 |
| Road | 0.091 | 0.149 | -0.200 | 0.383 |
| Water | 0.072 | 0.155 | -0.231 | 0.376 |
| *Breeding period 2012* |  |  |  |  |
| **Intercept** | **-1.351** | **0.184** | **-1.711** | **-0.992** |
| **DTurb** | **0.317** | **0.142** | **0.037** | **0.596** |
| **Sunrise^2** | **0.257** | **0.091** | **0.079** | **0.435** |
| **Road** | **0.364** | **0.119** | **0.130** | **0.598** |
| Auto | -0.176 | 0.376 | -0.914 | 0.562 |
| **Settle** | **-0.305** | **0.104** | **-0.510** | **-0.101** |
| Water | -0.159 | 0.161 | -0.474 | 0.156 |
| Forest | -0.192 | 0.137 | -0.459 | 0.076 |
| Sunrise | -0.087 | 0.053 | -0.191 | 0.017 |
| **NTurb** | **-0.091** | **0.018** | **-0.126** | **-0.057** |
| FieldSize | -0.101 | 0.170 | -0.434 | 0.232 |
| *Autumn migration 2011* |  |  |  |  |
| **Intercept** | **-2.796** | **0.528** | **-3.830** | **-1.761** |
| DTurb | 0.625 | 0.373 | -0.107 | 1.357 |
| Auto | 0.751 | 1.003 | -1.215 | 2.718 |
| Sunrise | -0.266 | 0.228 | -0.712 | 0.180 |
| Water | 0.210 | 0.350 | -0.477 | 0.897 |
| Settle | 0.181 | 0.360 | -0.525 | 0.886 |
| Forest | -0.186 | 0.378 | -0.926 | 0.555 |
| NTurb | -0.170 | 0.385 | -0.925 | 0.585 |
| *Autumn migration 2012* |  |  |  |  |
| **Intercept** | **-4.591** | **1.180** | **-6.903** | **-2.279** |
| Settle | 1.180 | 0.669 | -0.132 | 2.492 |
| NTurb | -0.955 | 0.649 | -2.227 | 0.317 |
| **Road** | **2.088** | **0.766** | **0.586** | **3.590** |
| Auto | 1.324 | 2.396 | -3.373 | 6.021 |
| Sunrise | -0.455 | 0.442 | -1.320 | 0.411 |
| CV | -0.780 | 0.827 | -2.401 | 0.840 |
| Forest | 0.883 | 0.678 | -0.445 | 2.211 |
| Water | 0.316 | 0.399 | -0.466 | 1.097 |
| DTurb | -0.989 | 0.551 | -2.069 | 0.091 |
